# Ultrastructural characterization of dark microglia during aging in a mouse model of Alzheimer’s disease pathology and in human post-mortem brain samples

**DOI:** 10.1186/s12974-022-02595-8

**Published:** 2022-09-27

**Authors:** Marie-Kim St-Pierre, Micaël Carrier, Fernando González Ibáñez, Eva Šimončičová, Marie-Josée Wallman, Luc Vallières, Martin Parent, Marie-Ève Tremblay

**Affiliations:** 1 Axe Neurosciences, Centre de Recherche du CHU de Québec-Université Laval, Québec, QC Canada; 2 Department of Molecular Medicine, Université Laval, Québec City, QC Canada; 3 Division of Medical Sciences, University of Victoria, Victoria, BC Canada; 4 Département de Psychiatrie et de Neurosciences, Faculté de Médecine, Université Laval, Quebec, QC Canada; 5 Neuroscience Graduate Program, University of Victoria, Victoria, BC Canada; 6 CERVO Brain Research Center, Quebec, QC Canada; 7 Department of Biochemistry and Molecular Biology, The University of British Columbia, Vancouver, BC Canada; 8 Department of Neurology and Neurosurgery, McGill University, Montréal, QC Canada; 9 Centre for Advanced Materials and Related Technology (CAMTEC), University of Victoria, Victoria, BC Canada

**Keywords:** Microglia, Dark microglia, Ultrastructure, Alzheimer’s disease, Human post-mortem brain samples, Dystrophic neurites, Amyloid-beta

## Abstract

A diverse heterogeneity of microglial cells was previously described in Alzheimer’s disease (AD) pathology, including dark microglia, a state characterized by ultrastructural markers of cellular stress. To provide novel insights into the roles of dark microglia during aging in the context of AD pathology, we performed a quantitative density and ultrastructural analysis of these cells using high-throughput scanning electron microscopy in the ventral hippocampus CA1 *stratum lacunosum-moleculare* of 20-month-old APP-PS1 vs C57BL/6J male mice. The density of dark microglia was significantly higher in APP-PS1 vs C57BL/6J mice, with these cells accounting for nearly half of all microglia observed near amyloid-beta (Aβ) plaques. This dark microglial state interacted more with dystrophic neurites compared to other APP-PS1 microglia and possessed glycogen granules, associated with a metabolic shift toward glycolysis, which provides the first ultrastructural evidence of their presence in microglia. Dark microglia were further observed in aging human post-mortem brain samples showing similar ultrastructural features as in mouse. Overall, our results provide a quantitative ultrastructural characterization of a microglial state associated with cellular stress (i.e., dark microglia) that is primarily restricted near Aβ plaques and dystrophic neurites. The presence of this microglial state in the aging human post-mortem brain is further revealed.

**Supplementary Information:**

The online version contains supplementary material available at 10.1186/s12974-022-02595-8.

## Introduction

Alzheimer’s disease (AD) is a neurodegenerative disease associated with aging, which is characterized by the accumulation of intracellular neurofibrillary tangles (NFT) consisting of hyperphosphorylated tau and extracellular amyloid-beta (Aβ), which can form fibrillar Aβ plaques [1]. These pathological signs are associated with neuronal and synaptic loss, the latter representing one of the best correlates for cognitive decline across several brain regions, including the hippocampus, notably important for learning and memory as well as emotional regulation [1, 2]. Hippocampal atrophy is a common feature of AD that can be observed in other dementias (e.g., vascular, frontotemporal lobar) and during normal aging [3, 4]. The hippocampus is composed of several subregions, one of which, the CA1, is the first hippocampal area affected by pathological signs of AD, including the appearance of NFTs and Aβ plaques [5], as well as sustained (neuro)inflammation [6–9].

Recent genome-wide association studies (GWAS) have shown that numerous gene variants linked with a higher risk of developing late-onset AD (LOAD) [i.e., triggering receptor expressed on myeloid cells 2 (*trem2*), bridging integrator 1 (*bin*1), myeloid cell surface antigen CD33 (*cd33*), apoliprotein E (*apoe*)] affect the function of microglia [10–12], the resident immune cells of the central nervous system (CNS). Microglia have been shown to participate in the development of tau pathology [13], notably through synaptic spreading [14] and early on during disease progression in the compaction, formation and elimination of Aβ plaques across mouse models of AD pathology [15–17]. This evidence strongly suggest that this brain immune cell plays a crucial role in driving the pathogenesis of this neurodegenerative disease.

Microglia first originate from the yolk sac to colonize the CNS in mouse starting around embryonic day (E) 9.5 [18] corresponding to the 4–5th week of gestation in humans [19–21], after which they proliferate, migrate to their respective territory, and perform various physiological functions crucial for the development and maintenance of CNS homeostasis [22–26]. These cells possess highly dynamic processes that survey the parenchyma for insults and cues, both of endo- and exogenous natures [27, 28]. Beside their surveying functions, microglia are key players in synaptic plasticity, where they can release brain-derived neurotrophic factor promoting synapse formation [29], nibble (trogocytosis) [30] or phagocytose partially or entirely weaker synapses, and dissociate pre- and post-synaptic elements to modify synaptic connectivity (synaptic stripping) [31–33].

In pathological conditions, such as AD, microglial features and functions are altered, a change observed during the onset and progression of the disease. Various microglial states, a term defining microglial groups with distinct characteristics [34], were investigated in mouse models of AD pathology, including dystrophic microglia (also termed senescent microglia in the literature) which are positive for L-ferritin, possess lipofuscin deposits and distinct morphological features (i.e., spherical swellings in their tortuous processes) [35–39]. In addition, both in human samples [40–42] and mouse models of AD pathology [43], microglia agglomerate near Aβ plaques and accumulated dystrophic neurites, while in mouse models of AD pathology, they were shown to restrict plaque growth and reduce the overall amyloid load via their phagocytosis of extracellular Aβ [16, 17, 44–48]. However, later in the disease process, microglia become unable to perform their critical phagocytic functions properly, thereby resulting in increased pathology [12, 49–53].

While still under debate, plaque-associated microglia were shown to shift their metabolism to anaerobic glycolysis rather than oxidative phosphorylation, allowing these cells to quickly but less efficiently, generate the adenosine triphosphate (ATP) required for their functions [54–56]. This results in an increased production of reactive oxygen species (ROS) [57], which can structurally and functionally alter cellular organelles such as mitochondria and/or the endoplasmic reticulum (ER) [58–60]. In addition, previous work in 5xFAD mice, a model of AD pathology, and following Aβ exposure in cultured primary microglia from neonatal mice, identified that this metabolic switch in microglia was accompanied by a pro-inflammatory signature [54]. Minhas et al*.* further revealed that inhibiting this metabolic shift, associated with pro-inflammatory responses in the periphery and CNS, could reverse age-related cognitive decline in mice [61].

Beyond this metabolic alteration, a wide array of microglial states were recently uncovered in mouse models of AD pathology. The Aβ plaque-associated microglial signature was characterized by an increased expression of genes associated with LOAD (e.g., *inpp5d, trem2, apoe, tyrobp*) [11, 12]*.* These microglia, located near Aβ plaques, further exhibited a unique signature [e.g., disease-associated microglia (DAM) [62], neurodegenerative phenotype (MgND) [63], activated-response microglia (ARM) [11]], as well as diverse ultrastructural alterations [64, 65] that corroborate the incredibly varied nature of microglia described in AD pathology [43, 66]. The numerous microglial states observed in mouse models of AD pathology, which recreate either and/or the Aβ deposition and NFTs [67], also exhibit a similarly reduced expression of homeostatic genes (e.g., *tmem119, p2ry12, cx3cr1*). In line with these findings, previous studies from our group identified a microglial state, the dark microglia, which are found nearby fibrillar Aβ in middle-age, 14-month-old APP-PS1 mice [mouse model of AD with a Swedish mutation in the APP and a humanized presenilin (PSEN1)] [64, 65].

Dark microglia, which are immunonegative for the homeostatic microglial marker P2RY12 and weakly positive for CX3CR1 and Iba1, were identified based on their ultrastructural markers of cellular stress (e.g., condensed cyto- and nucleoplasm, dilated ER, altered mitochondria) distinguishing them from other microglia (refered hereafter as typical microglia). Dark microglia were found to interact extensively with blood vessels, synaptic elements (axon terminals and dendritic spines showing dystrophy) and display a strong immunoreactivity for cluster of differentiation molecule 11B (CD11b) and TREM2 [64], a receptor which appears crucial for the appearance of several disease-associated microglial states (DAM, MgND) [62, 63]. A previous study by Bisht et al*.* revealed that dark microglia can reach a density up to 34 cells per mm^2^ in the hippocampus CA1 of middle-age 14 month-old APP-PS1 male mice, compared to 11 cells per mm^2^ in age-matched controls and 3 cell per mm^2^ found in younger 3-month-old controls [64]. Their frequent interactions with dystrophic synaptic elements and the vasculature have suggested key roles in their remodeling, which are particularly exacerbated in AD pathology, hence resulting in synaptic loss and blood–brain barrier disruption [68, 69].

While dark microglia were previously observed near Aβ plaques and dystrophic neurites among the ventral hippocampus CA1 of APP-PS1 mice [64, 65], their relationship to AD hallmarks (Aβ plaques, dystrophic neurites), other elements of the CNS parenchyma, and their ultrastructural markers [i.e., dilated ER, altered mitochondria] remained to be quantitively analyzed. As aging is the main risk factor for developing LOAD [3], we studied microglial heterogeneity in 20-month-old male mice, a timepoint corresponding to an age range between 56 and 69 years in human individuals, a timeframe where most cases are diagnosed [3, 70]. In this study, we aimed to quantify the density, distribution and features of dark vs typical microglia based on their distance to Aβ plaques and dystrophic neurites in 20-month-old APP-PS1 male mice compared to age-matched C57BL/6J male mice. We compared aged APP-PS1 to age-matched C57BL/6J mice to determine the selective outcome of AD pathology on microglial ultrastructural heterogeneity, by excluding the effects that aging can have on the cellular state (e.g., cellular stress ultrastructurally shown by dilated ER and altered mitochondria). Our observations were conducted in the ventral hippocampus CA1 *stratum lacunosum-moleculare*, where we previously identified dark microglia [64], using high-resolution scanning electron microscopy (SEM). Dark microglia were almost exclusively localized nearby Aβ plaques and dystrophic neurites in aged APP-PS1 mice contrary to age-matched C57BL/6J controls. Moreover, we found that the dark microglia interacted more frequently with dystrophic vs non-dystrophic axon terminals. While dark microglia often displayed altered mitochondria and dilated ER/Golgi apparatus, both the cells’ distribution and mouse genotype accounted for their prevalence of these ultrastructural markers of cellular stress. Glycogen granules, previously not identified in microglia [71, 72], were further observed in the cytoplasm of both typical and dark microglia located nearby Aβ plaques and dystrophic neurites, with a higher prevalence encountered in dark microglia. Finally, we confirmed for the first time the presence of dark microglia displaying similar features as their mouse counterparts in the parahippocampal gyrus and hippocampal head of middle-aged and aged human post-mortem brain samples.

## Methods

### Animals and mouse brain tissue cutting

All experiments were performed according to the guidelines of the Institutional Animal Ethics Committees, the Canadian Council on Animal Care, as well as the Animal Care Committee of Université Laval. Four, 8 and 20-month-old C57BL/6J and APP^Swe^-PS1Δe9 male mice (No. 34832-JAX, Jackson Laboratory, Maine, USA) (*n* = 4) on a C57BL/6J background [73] were housed under a 12 h light–dark cycle at 22–25 °C with ad libitum access to food and water. While AD affects both males and females (with females being at higher risk notably due to their increased longevity [74]), we performed the experiments on males as this sex was previously used to investigate the presence of dark microglia in 14-month-old APP-PS1 and C57BL/6J mice [64]. The experimental mice were injected with 10 g/kg Methoxy-X04 (Tocris Bioscience, cat# 4920, Bristol, United Kingdom), allowing for the screening of fibrillar Aβ with fluorescence microscopy [75]. For electron microscopy experiments, mice were anesthetized 24 h later by i.p. injection of sodium pentobarbital (80 mg/kg), followed by transcardial perfusion using 3.5% acrolein [diluted in phosphate buffer (PB): 100 mM at pH 7.4] and 4% paraformaldehyde [PFA, diluted in phosphate-buffered saline (PBS): 50 mM at pH 7.4]. Fifty-micrometer thick coronal brain sections were then generated in ice-cold PBS using a vibratome (Leica VT1000S) and kept at -20˚C in cryoprotectant solution [20% (v/v) glycerol and 20% (v/v) ethylene glycol in PBS] until further experimentation [76].

### Human brain tissue cutting and immunostaining

Coronal hippocampal sections containing the entorhinal cortex, hippocampal head, CA1, CA2 and parahippocampal gyrus from middle-aged and aged individuals (male, 45 years old, cause of death—acute pulmonary edema; female, 81 years old, cause of death—asphyxia; both 18 h post-mortem interval) were obtained from the brain bank located at the CERVO Brain Research Center (QC, Canada). The brain bank and handling of the post-mortem human tissues were approved by the Ethics Committee of the Institut Universitaire en Santé Mentale de Québec and Université Laval. Written consent was obtained for the use of post-mortem tissues and all analyses were performed in line with the Code of Ethics of the World Medical Association. Brains were sectioned along the midline and hemibrains were cut coronally into 2-cm thick samples. The latter were incubated 3 days at 4 °C in 4% PFA and stored at 4 °C in PBS with 15% sucrose and 0.1% sodium azide. Regions of interest were subsequently cut into 50 µm-thick sections using a vibratome and stored at -20˚C in cryoprotectant solution until immunostaining [65].

For immunostaining, human brain sections containing the regions of interest (CA1, CA2, parahippocampal gyrus, enthorinal cortex, hippocampal head) were first washed in PBS and quenched for 5 min in 0.3% H_2_O_2_ diluted in PBS. Following the quenching step, sections were washed 3 times 10 min in PBS and incubated for 30 min in 0.1% NaBH_4_ diluted in PBS. After washing 3 times 10 min in PBS, the sections were blocked in a solution containing 10% fetal bovine serum, 3% bovine serum albumin and 0.01% Triton X-100 [in Tris-buffered saline (TBS), 50 mM, pH 7.4] for 1 h. The sections were then incubated overnight at 4 °C in primary rabbit antibody against ionized calcium binding adaptor molecule 1 (Iba1, FUJIFILM Wako Chemical Virginia, USA, cat# 019-19741) diluted 1/1000 in the blocking buffer. The following day, the sections were washed 3 times 10 min in TBS and incubated in biotinylated goat anti-rabbit secondary antibody (1/300 in TBS, Jackson Immunoresearch, Philadelphia, USA, cat# 111-066-046). After 3 times 10 min washes in TBS, the sections were incubated for 1 h in an avidin–biotin solution (1/100 in TBS; VECTASTAIN^®^, Vector Labs, California, USA, cat# VECTPK6100) and washed 3 times 10 min in TBS. The staining was then revealed in a solution containing 0.05% 3-3′-diaminobenzidine (DAB, Millipore Sigma, MA, USA, D5905-50TAB) and 0.015% H_2_O_2_ in 100 mM Tris–HCl.

### Mouse and human sample preparation for electron microscopy and imaging

Brain sections containing the ventral hippocampus (Bregma -2.92 to 3.64 mm) from 4, 8 and 20-month-old APP-PS1 and age-matched C57BL/6J mice (*n* = 3–4), as well as immunostained post-mortem human samples containing the regions of interest (CA1, CA2, parahippocampal gyrus, entorhinal cortex, hippocampal head) were processed for SEM. The protocol for the sample preparation was recently detailed in St-Pierre et al. [77]. Briefly, the sections were washed in PB and incubated 1 h in a solution containing equal volume of 4% osmium tetroxide (EMS, Pensylvannia, USA, cat# 19190) and 3% potassium ferrocyanide (Sigma-Aldrich, Ontario, Canada, cat# P9387) in PB. After washing in PB, the sections were incubated 20 min in a filtered 1% thiocarbohydrazide solution (diluted in MilliQ water; Sigma-Aldrich, Ontario, Canada, cat# 223220) followed by a second 30 min incubation with 2% aqueous osmium tetroxide (diluted in MilliQ water). Once the sections were post-fixed, they were dehydrated in ascending concentrations of ethanol for 10 min each (2 × 35%, 50%, 70%, 80%, 90%, 3 × 100%) and washed 3 times for 10 min with propylene oxide (Sigma-Aldrich, #cat 110205-18L-C). The tissues were then embedded in Durcupan resin (20 g component A, 20 g component B, 0.6 g component C, 0.4 g component D; Sigma Canada, Toronto, cat# 44610) for 24 h. The following day, for flat-embedding, the resin-infiltrated brain sections were placed onto fluoropolymer films (ACLAR^®^, Pennsylvania, USA, Electron Microscopy Sciences, cat# 50425-25) painted with resin and kept at 55 °C in a convection oven for 3 days to allow for resin polymerization.

After resin polymerization, areas containing the regions of interest from the mouse and human samples were excised and glued onto resin blocks for ultramicrotomy sectioning. Using a Leica ARTOS 3D ultramicrotome, 70-nm thick sections from these areas were cut (2–6 levels, ~ 5–6 µm apart) and collected on silicon wafers for imaging by SEM or on copper mesh grids for transmission electron microscopy imaging. With SEM, pictures were acquired at a resolution of 25 nm for the density analysis and 5 nm of resolution for the ultrastructural analysis of microglia using a Zeiss Crossbeam 540 SEM, operating at a 1.4 kV voltage and 1.2 nA current. The software Zeiss Atlas 5 (Fibics, Ottawa) was used to stitch and export the images in.tif format. Sections from 4-month-old APP-PS1 and C57BL/6J male mice were additionally screened for dark microglia’s presence using a JOEL JEM-1400 transmission electron microscope operated at 80 kV and microglia were imaged with a Gatan SC-1000 digital camera at 6000× magnification.

### Quantitative analysis of typical vs dark microglia in APP-PS1 and C57BL/6J mice

For the density analysis in mouse samples, images from 4 animals per group captured in the CA1 *stratum lacunosum-moleculare* were blinded to the experimental condition to avoid introducing bias. In each animal, 2–6 levels (~ 5–6 µm apart) were analyzed to determine the average density of different microglial states. Typical and dark microglia, identified by their ultrastructural features detailed below, were further categorized based on their distance to fibrillar Aβ plaques and dystrophic neurites. Microglia were determined to be near an Aβ plaque if the most proximal point of their cell body was less than 25 µm away from the plaque core. The density of typical and dark microglia, as well as the ratio of dark microglia over all microglia imaged, based on their distance from AD hallmarks, was calculated in 25 nm resolution pictures. Dark microglia were differentiated from typical microglia by their electron-dense cyto- and nucleoplasm and a distinctive loss of the nuclear heterochromatin pattern [64, 65, 76, 78]. Intermediate microglia were identified by the presence of a less-defined heterochromatin pattern and dark cytoplasm similar to the dark microglia, and their markers of cellular stress, such as ER dilation and altered mitochondria [65, 77]. Intermediate and dark microglia were pooled for the density and ultrastructural analyses of dark microglia as both cell states present a condensation of their nucleo- and cytoplasm as well as ultrastructural markers of cellular stress [65].

For the ultrastructural analysis, 9–12 microglia per animal per localization to plaques (far or near), state (typical or dark) and genotype (C57BL/6J vs APP-PS1) were imaged at 5 nm of resolution within the CA1 stratum *lacunosum-moleculare*, where dark microglia were previously found [64, 65]. The images were blinded to the experimental condition to prevent bias. To quantify ultrastructural changes, we analyzed 111 microglial cell bodies in total (27–29 microglia per condition), a sample size which was considered sufficient to obtain statistical power based on the G*Power software V3.1 (effect size of 0.4 and power of 0.95 estimated to 112 individual cells). A similar effect size was previously used to quantitatively assess microglial ultrastructure [79]. To compare dark vs typical microglia’s interactions with AD hallmarks (dystrophic neurites, Aβ plaques), we analyzed 55 microglial cell bodies (27–28 microglia per condition) in the *stratum lacunosum-moleculare*, which was considered suffient to obtain a large effect size of 0.9 using G*Power software V3.1 (power of 0.9) [80]. No immunostaining was performed to allow the experimenter to observe the electron density of the cytoplasm/nucleoplasm and examine the presence or absence of glycogen granules. The identification of microglia and their cytoplasmic content was previously described in detail [81]. Typical microglia were identified by their heterochromatin pattern and differentiated from oligodendrocytes by their long and narrow ER stretches, and presence of diverse inclusions (e.g., lysosomes, lipofuscin granules) dispersed heterogeneously in their cytoplasm [81]. In addition to the ultrastructural characteristics examined at 25 nm of resolution, the presence of oxidative stress markers (e.g., altered mitochondria, dilated ER) were further used to identify dark and intermediate microglia at 5 nm of resolution [64, 76].

Lysosomes were identified by their circular and homogenous (primary) or heterogenous (secondary, tertiary) content. Secondary lysosomes were larger than primary lysosomes and often contained empty phagosomes. Tertiary lysosomes were the largest and possessed large lipid bodies in addition to phagosomes [65, 82]. Lipid bodies, either contained in a lysosome or within the cytoplasm, were characterized by their electron-dense circular outline with a homogenous and electron-dense interior. Lipofuscin granules were identified by their electron-dense content with a unique fingerprint-like pattern. Fully digested (electron-lucent) or partially digested (electron-lucent with cellular content) phagosomes were identified by their defined circular shape and electron-lucent interior. Autophagosomes presented as double-membrane endosomes with a circular shape and an interior with a similar appearance to the cell’s cytoplasm [76, 77, 81, 83].

Fibrillar contents, which were previously described in [64, 65], are characterized by their elongated shape and fibrils, with electron-dense patches [84, 85]. Dystrophic neurites (axon terminals, dendrites and dendritic spines) contacted by the microglial cell bodies were characterized by an accumulation of electron-dense autophagic vacuoles and mitochondria, together with a swollen appearance. Fibrillar Aβ plaques associated with microglial cell bodies were identified by their heterogenous fibrils forming a tree-like shape [75, 77]. Myelinated axons were identified by their electron-dense sheaths and granular cytoplasm often containing mitochondria [86]. Extracellular digestion and extracellular space consisted of electron-lucent space devoid of a membrane delineation located next to a microglia and containing (digestion) or not (space) partially-digested cellular elements [87]. Axon terminals were defined by their numerous synaptic vesicles, while dendritic spines were recognized by their postsynaptic density [83, 88]. Microglial contacts with synaptic elements were categorized as axon terminals, dendritic spines or both (when the two elements and synaptic cleft were contacted simultaneously) [77]. Microglia were considered to be associated with the vasculature if the distance between their cell body plasma membrane and the vascular basement membrane was under 150 nm (justavascular microglial cell soma were previously considered to be associated with the vasculature when closer to 10 µm using in vivo 2-photon microscopy [89]).

Endoplasmic reticulum cisternae were characterized by their long and narrow stretches found in the microglial cytoplasm [81]. Dilated ER and Golgi apparatus were identified by the swollen electron-lucent appearance of these organelles (determined as dilated if the cisternae diameter was over 100 nm) [77]. Mitochondria were identified by their electron-dense appearance, double membrane, numerous cristae, and circular shape. Altered mitochondria were characterized by a deterioration of the outer membrane, degradation of the cristae (electron-lucent pockets) or “holy” shape (i.e., mitochondria forming a donut shape) [76]. Mitochondria were defined as elongated if their length was over 1000 nm [90]. Glycogen granules were identified by their electron-dense, small and rounded shape with a diameter of 22–40 nm. This is in line with the reported literature which identified β-granules in the brain (glycogen granules comprise several γ-granules which are termed β-granules) at a maximum size of 42 nm, while the average size is approximately 20–30 nm [91]. Microglia containing more than 5 of these granules were determined to be positive to prevent a false-positive identification.

To assess microglial area, perimeter and morphology, the outline of each microglial cell body was traced using the freehand tool in Image J and the shape descriptors i.e., aspect ratio, area, perimeter, solidity, and circularity were assessed. Aspect ratio (AR) refers to the ratio of the height over the width of the microglial cell body. Solidity is calculated by dividing the area of the cell over the convex area and is an indicator of irregular shapes. The closer the value of the solidity is to 1, the less irregular shape the microglial cell body possesses [86, 92]. Circularity is calculated as 4π times the area over the perimeter squared. Circularity is an indicator of the cell’s shape, where a value closer to 1 indicates a rounder shape and a value near 0 reflects a more elongated shape [65, 86, 93].

### Qualitative analysis of typical vs dark microglia in the human brain samples

Similar to microglia in mouse brains, typical microglial cell bodies in human post-mortem brain samples were identified based on their heterochromatin pattern, presence of lipofuscin deposits and a positive immunoreactivity to Iba1. Dark microglia were further identified based on their electron-dense cyto- and nucleoplasm, while they possessed in these samples an heterochromatin pattern similar to typical microglia. Their cellular content (e.g., phagosomes, mitochondria, lysosomes, lipofuscin granules, ER, Golgi apparatus) and interactions with the CNS parenchyma (axon terminals, dendritic spines, myelinated axons, blood vasculature) were assessed and found to be similar to those in mouse microglia.

### Statistical analysis

Prism 9 (v.9.2.0, GraphPad software) was used for statistical analysis in mice. All density and ultrastructure data obtained were tested for their normality using the Shapiro–Wilk test. The quantitative ultrastructural and density comparison of typical microglia in C57BL/6J mice, typical microglia far from plaques in APP-PS1 mice, as well as typical and dark microglia near plaques of APP-PS1 mice was performed with a Kruskal–Wallis One-Way ANOVA followed by a Dunn’s multiple comparison *post-hoc* test. The interactions of typical and dark microglia with AD pathology hallmarks was compared with a Mann–Whitney non-parametric test. Data are expressed as mean ± standard error of the mean (SEM). The sample size (n) refers to individual animals for density analysis and individual microglia for ultrastructural analyses as previously performed in ultrastructural studies of microglia [65, 82, 86, 89, 90, 94, 95]. Statistically significant differences are reported as *p < 0.05, **p < 0.01, ***p < 0.001 and, ****p < 0.0001.

## Results

### Dark microglia are highly prevalent in aged APP-PS1 mice compared to age-matched C57BL/6J mice

Dark microglia were previously observed in the ventral CA1 *stratum lacunosum-moleculare* of middle-age 14-month-old APP-PS1 mice, where they were localized nearby Aβ plaques and dystrophic neurites [64, 65, 78]. These cells were ultrastructurally defined by their condensed electron-dense cyto- and nucleoplasm, accompanied by several markers of oxidative stress (e.g., ultrastructurally altered mitochondria, dilated ER cisternae) [64, 76]. To investigate and characterize dark microglia during aging, the main risk factor for AD [96], we first determined their distribution using high magnification SEM chip mapping, within the ventral CA1 *stratum lacunosum-moleculare*, which exhibits considerable atrophy in APP-PS1 mice and patients with AD [4, 97], and where dark microglia were previously shown to be abundant in 14-month-old C57BL/6J and APP-PS1 mice [64]. In particular, the density of dark vs typical microglia was determined among areas associated with Aβ plaques and dystrophic neurites. Typical microglia were previously shown to exhibit ultrastructural diversity based on their proximity to dystrophic neurites [65]. Previous studies have also shown the association of various microglial states (dark microglia, DAM, MGnD) with Aβ plaques and dystrophic neurites [62–64].

**Fig. 1:**
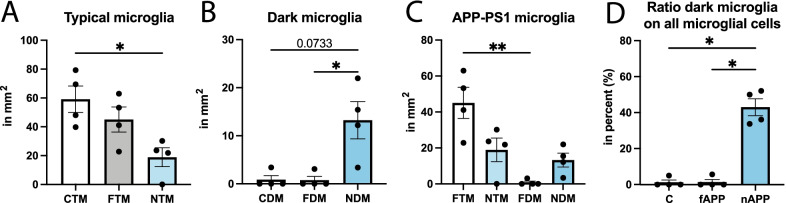
Distribution and density of dark vs typical microglia. Density analysis based on ultrastructural characteristics of dark (DM) and typical microglia (TM) in the ventral hippocampus CA1 *stratum lacunosum-moleculare* of 20-month-old and age-matched C57BL/6J male mice. The density of TM (**A**) and DM (**B**) is shown. The density of microglia based on their proximity to plaques (far or near) as well as their state (TM or DM) is provided (**C**). The percentage of DM over all the microglia far from plaques or near plaques is represented in **D**. Data shown are expressed as means ± S.E.M. *p < 0.05, **p < 0.01, using a Kruskal–Wallis test with a post hoc Dunn’s multiple comparisons test. n = 4 animals

In the *stratum lacunosum-moleculare*, the density of typical microglia near Aβ plaques and dystrophic neurites (NTM) was significantly reduced compared to control C57BL/6J typical microglia (CTM) (NTM 18.91 ± 6.555 mm^2^ vs CTM 59.13 ± 9.170 mm^2^, *p* = 0.0427), while no difference was observed for typical microglia located far from Aβ plaques and dystrophic neurites (FTM) (Fig. 1A). Conversely, the density of dark microglia was significantly greater near Aβ plaques and dystrophic neurites (NDM) than far from Aβ plaques and dystrophic neurites (FDM) (NDM 13.24 ± 3.827 mm^2^ vs FDM 0.7747 ± 0.7747 mm^2^,* p* = 0.0417). A similar trend was observed for dark microglia in age-matched C57BL/6J mice (CDM) (NDM 13.24 ± 3.827 mm^2^ vs CDM 0.8559 ± 0.8559 mm^2^, *p* = 0.0733) (Fig. 1B). When comparing the density of typical and dark microglia in the APP-PS1 mice based on their distribution near Aβ plaques and dystrophic neurites, we observed significantly more typical microglia compared to dark microglia far from Aβ plaques and dystrophic neurites (FTM 45.04 ± 8.654 mm^2^ vs FDM 0.7747 ± 0.7747 mm^2^, *p* = 0.0045), while no difference was observed for dark and typical microglia near these two AD pathology hallmarks (Fig. 1C). Dark microglia represented nearly 43% of all microglia found near Aβ plaques and dystrophic neurites (Fig. 1D), while the percentages were reduced to 1.4% and 1.2%, respectively, for dark microglia located far from Aβ plaques and dystrophic neurites and those observed in age-matched C57BL/6J mice.

### Dark microglia interact more with dystrophic neurites compared to their typical counterpart in aged APP-PS1 mice

Our results indicate that dark microglia preferentially associate with hallmarks of neurodegeneration in the ventral CA1 *stratum lacunosum-moleculare* of 20-month-old APP-PS1 mice. This specific localization is corroborated by our previous studies where dark microglia were often localized near Aβ plaques in the ventral CA1 *strata lacunosum-moleculare* and *radiatum* of 14-month-old APP-PS1 mice [64, 65]. In the current study, we found in the ultrathin sections imaged by SEM that 52.5% of Aβ plaques were associated with at least one dark microglia within a perimeter of 25 µm. It is likely that the proportion of dark microglia juxtaposing plaques could be higher but was underestimated due to the thinness of the samples(70 nm). To provide further insights into the interactions between Aβ plaques, dystrophic neurites and dark microglia, we examined two earlier timepoints, i.e., 4 months of age when plaques have started to develop and are yet few in numbers [98, 99] and 8 months of age when plaques have appeared in this model. In these mice, we confirmed the presence of Aβ plaques by systemically injecting Methoxy-X04, a fluorescent derivative of congo red that stains Aβ [75], 24 h prior to the euthanasia, as well as their distinct ultrastructure in SEM [77]. We did not identify dark microglia in the 4-month-old APP-PS1 mice devoid of Aβ plaques and dystrophic neurites, confirming that a significant presence of pathological signs related to Aβ deposition is crucial for the appearance and accumulation of these cells. By contrast, in the 8-month-old APP-PS1 mice, we observed a similar pattern as in 20-month-old mice, where dark microglia were localized near Aβ plaques and dystrophic neurites (Additional file 1: Fig. S1).

**Fig. 2:**
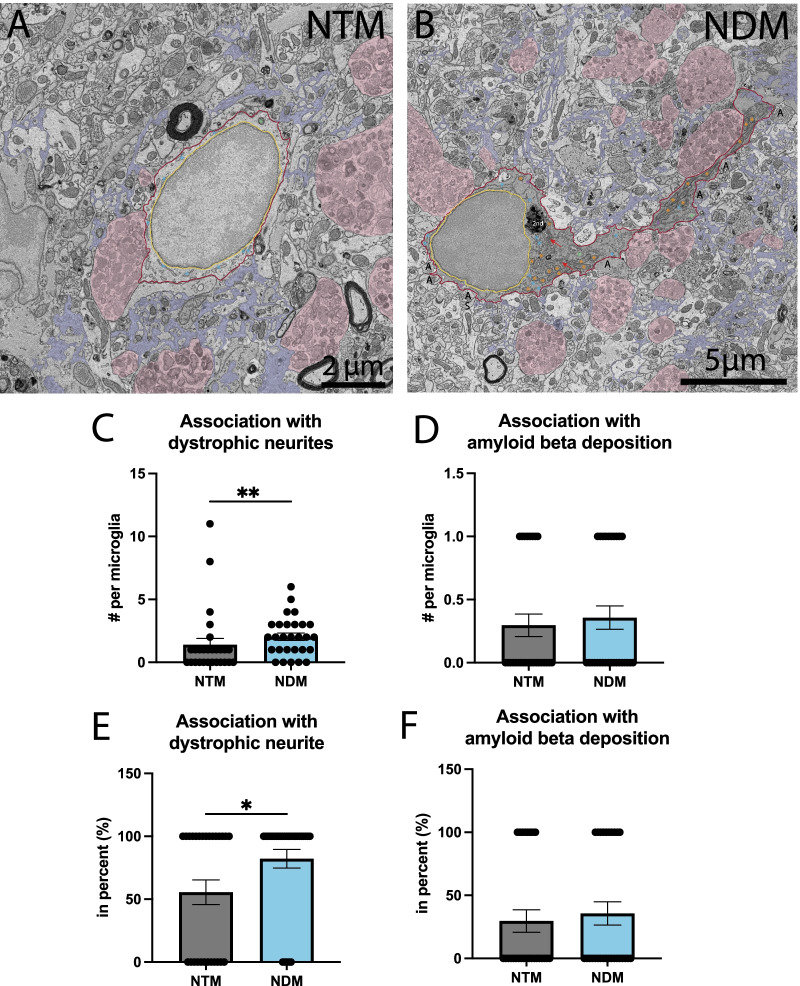
Dark vs typical microglia’s interactions with dystrophic neurites and Aβ plaques. Representative 5 nm resolution scanning electron microscopy images captured in the ventral hippocampus CA1 *stratum lacunosum-moleculare* of 20-month-old APP-PS1 male mice. **A** Typical microglia (TM) observed near extracellular fibrillar Aβ (pseudocolored in purple) and dystrophic neurites (pseudocolored in pink). **B** Dark microglia (DM) interacting with several dystrophic neurites along with fibrillar Aβ. **C**–**F** Quantitative graphs representing the numbers of contacts with dystrophic neurites (**C**) and Aβ plaques (**D**) as well as the proportion of microglial cells contacting dystrophic neurites (**E**) or Aβ plaques (**F**). Data are shown as individual dots of either 0 or 100 values and are expressed as means ± S.E.M. * p < 0.05, ** p < 0.01, using a non-parametric Mann–Whitney test. Statistical tests were performed on n = 9–12 microglia per animal with N = 3 mice/group, for a total of 111 microglial cell bodies analyzed. Purple pseudo-coloring = fibrillar Aβ, pink pseudo-coloring = dystrophic neurites, red outline = plasma membrane, yellow outline = nuclear membrane, orange asterisk = mitochondria, green asterisk = altered mitochondria, blue asterisk = endoplasmic reticulum, purple asterisk = dilated endoplasmic reticulum, red arrow = Golgi apparatus, 2nd = secondary lysosome, A = axon terminal

Having shown the necessity of Aβ plaques and dystrophic neurites for the appearance of dark microglia in the APP-PS1 mice, we decided to further explore the direct interaction of dark vs typical microglia with Aβ plaques and dystrophic neurites. Dark microglia interacted more with dystrophic neurites than typical microglia (NDM 2.036 ± 0.2931 contact per microglia vs NTM 1.407 ± 0.4929 contact per microglia, *p* = 0.0045) (Fig. 2C). More dark microglia compared to typical microglia also directly interacted with at least one dystrophic neurite (NDM 82.14 ± 7.371% vs NTM 55.56 ± 9.745%, *p* = 0.0080) (Fig. 2C, E). Dark microglia and typical microglia made similar numbers of direct contacts with Aβ plaques (Fig. 2D, F). However, both typical and dark microglia directly touching an Aβ plaque contained fibrillar Aβ in their cytoplasm, suggesting that the two microglial states actively cooperate in contributing to reducing the Aβ load.

### Dark microglia interact less with parenchymal elements and the vasculature than typical microglia in aged APP-PS1 mice

Dark microglia contacted significantly more dystrophic neurites than typical microglia in 20-month-old APP-PS1 mice. To provide insights into the relationship between dark microglia and the CNS parenchyma, we next investigated their direct interactions with apparently healthy, or not, exhibiting ultrastructural signs of dystrophy, synaptic elements (axon terminals, dendritic spines), myelinated axons and the vasculature of APP-PS1 mice.

**Fig. 3:**
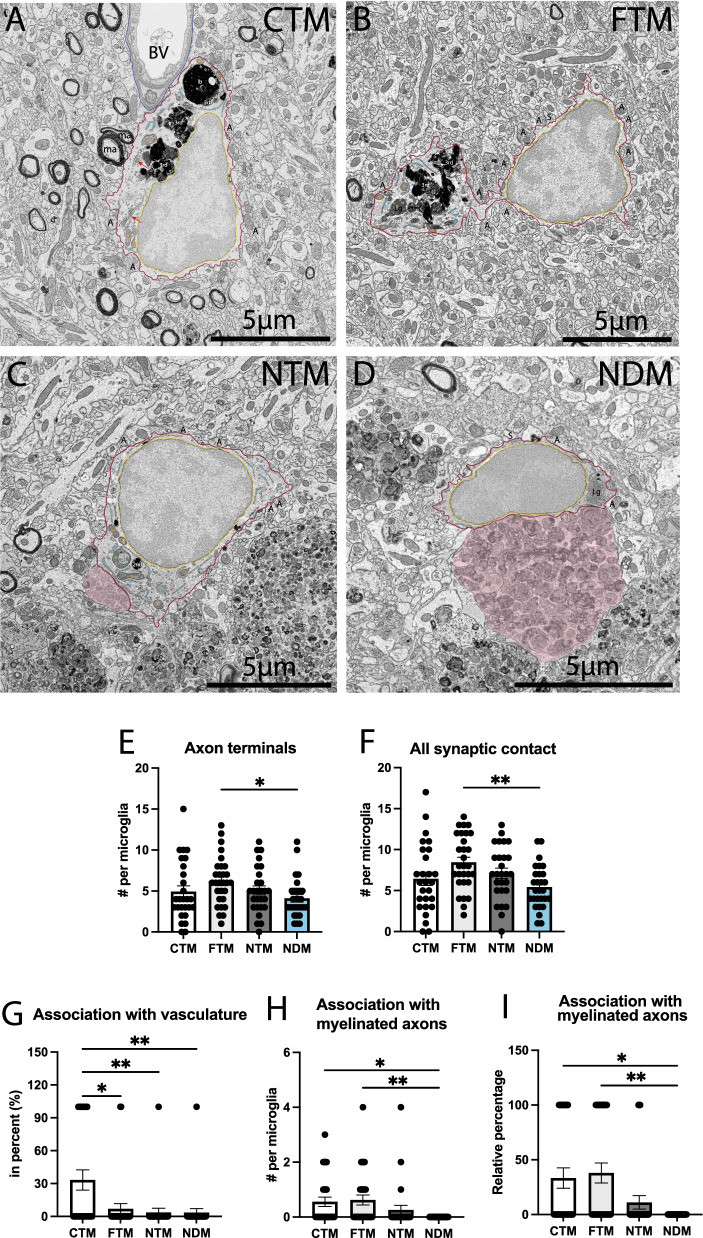
Dark vs typical microglia’s interactions with parenchymal elements. Representative 5 nm resolution scanning electron microscopy images taken in the ventral hippocampus CA1 *stratum lacunosum-moleculare* of 20-month-old APP-PS1 and C57BL/6J male mice. **A** Typical microglia (TM) in C57BL/6J mice contacting a blood vessel (labeled BV) and myelinated axons (labeled ma) as well as axon terminals (labeled A), **B** TM far from a plaque interacting with axon terminals and dendritic spines (labeled S), **C** TM near a plaque interacting with a few axon terminals, **D** dark microglia (DM) near a plaque is contacting axon terminals. Graphs representing the number of axon terminals (**E**), all synaptic interactions (**F**), percentage of cells associating with the vasculature (**G**), myelinated axons (**F**), as well as the percentage of cells touching a myelinated axon (**J**). Data shown are expressed as means ± S.E.M. * p < 0.05, ** p < 0.01, using a Kruskal–Wallis test with a Dunn’s multiple comparisons post hoc test. Statistical tests were performed on n = 9–12 microglia per animal with N = 3 mice/group, for a total of 111 microglial cell bodies analyzed. Red outline = plasma membrane, yellow outline = nuclear membrane, blue outline = basement membrane, ma = myelinated axons, A = axon terminals, S = dendritic spines, orange asterisk = mitochondria, green asterisk = altered mitochondria, blue asterisk = endoplasmic reticulum, red arrow = Golgi apparatus, lb = lipid body, 3^rd^ = tertiary lysosome, 2nd = secondary lysosome, Lg = lipofuscin granules, pink pseudo-coloring = dystrophic neurites

In terms of synaptic relationships, dark microglia located near Aβ plaques and dystrophic neurites contacted significantly less non-dystrophic elements (axon terminals, dendritic spines, both elements of the same excitatory synapse) than typical microglia located far from Aβ plaques and dystrophic neurites (NDM 5.429 ± 0.4945 contact per microglia vs FTM 8.448 ± 0.6250 contact per microglia, *p* = 0.0064). Specifically, this change was associated with reduced interactions with axon terminals of dark microglia located near Aβ plaques and dystrophic neurites compared to typical microglia located far from Aβ plaques and dystrophic neurites (NDM 4.107 ± 0.4581 contact per microglia vs FTM 6.207 ± 0.5490 contact per microglia, *p* = 0.0303) (Fig. 3E, F). However, this difference was not observed between dark microglia near Aβ plaques and dystrophic neurites, typical microglia near AD pathology hallmarks, and typical microglia in C57BL/6J mice (Tables 1, 2). This change could be due to dark microglia preferentially interacting more with dystrophic neurites vs non-dystrophic synaptic elements, highlighting a key difference in functional interventions based on the microglial state.

**Table 1:** Absolute ultrastructural analysis of dark microglia near Aβ plaques and dystrophic neurites compared to typical microglia near vs far Aβ plaques and dystrophic neurites in the ventral hippocampus CA1 *stratum lacunosum-moleculare* of aged-matched APP-PS1 vs C57BL/6J male mice

	C57BL/6J	APP-PS1		
	CTM Mean ± SEM (Min–Max)	FTM Mean ± SEM (Min–Max)	NTM Mean ± SEM (Min–Max)	NDM Mean ± SEM (Min–Max)
Area (µm^2^)	26.54 ± 1.702 (11.68–44.92)	28.20 ± 1.859 (11.32–45.09)	27.96 ± 2.094 (11.63–46.09)	26.76 ± 1.678 (10.30–49.48)
Perimeter (µm)	25.25 ± 1.437 (15.85–50.27)	24.54 ± 1.221 (14.60–42.87)	25.71 ± 1.837 (13.50–48.10)	32.49 ± 3.204 (5.758–68.19)
Circularity (a.u)~~	0.5545 ± 0.02709 (0.1900–0.8040)	0.5993 ± 0.02212 (0.2970–0.7850)	0.5820 ± 0.03226 (0.2350–0.8080)	0.4417 ± 0.03181**## (0.1550–0.6050)
Aspect ratio (a.u)	1.732 ± 0.08945 (1.035–2.765)	1.522 ± 0.07346 (1.085–2.635)	1.716 ± 0.109 (1.024–3.243)	1.992 ± 0.1596 (1.164–5.157)
Roundness (a.u)	0.6175 ± 0.03100 (0.3620–0.9660)	0.6942 ± 0.02864 (0.3810–0.9210)	0.6386 ± 0.03525 (0.3080–0.9770)	0.5690 ± 0.03428 (0.1940–0.8590)
Solidness (a.u) ~	0.8670 ± 0.01766 (0.5410–0.9710)	0.8891 ± 0.01269 (0.6420–0.9570)	0.8620 ± 0.02242 (0.6030–0.9730)	0.8038 ± 0.02264*# (0.4630–0.9530)
Associations with myelinated axons (n) ~ ~	0.5556 ± 0.1716 (0.000–3.000)	0.6207 ± 0.1818 (0.000–4.000)	0.2593 ± 0.1653 (0.000–4.000)	0.000 ± 0.000##! (0.000–0.000)
Axon terminals (n) ~	4.926 ± 0.7059 (0.000–15.00)	6.207 ± 0.5490 (1.000–13.000)	5.074 ± 0.5493 (0.000–11.00)	4.107 ± 0.4581# (1.000–10.00)
Dendritic spines (n)	0.4444 ± 0.1541 (0.000–3.000)	1.103 ± 0.2866 (0.000–5.000)	0.8889 ± 0.1631 (0.000–3.000)	0.4643 ± 0.1204 (0.000–2.000)
Synaptic clefts (n)	1.074 ± 0.2611 (0.000–6.000)	1.138 ± 0.2089 (0.000–4.000)	1.148 ± 0.1826 (0.000–3.000)	0.8571 ± 0.2557 (0.000–6.000)
All synaptic contacts (n) ~ ~	6.444 ± 0.8136 (0.000–17.00)	8.448 ± 0.6250 (2.000–14.00)	7.1111 ± 0.6300 (0.000–13.00)	5.429 ± 0.4945## (1.000–11.00)
Primary lysosomes (n)	0.000 ± 0.000 (0.000–0.000)	0.03448 ± 0.03448 (0.000–1.000)	0.07407 ± 0.05136 (0.000–1.000)	0.07143 ± 0.04956 (0.000–1.000)
Secondary lysosomes (n)	0.03704 ± 0.03704 (0.000–1.000)	0.1034 ± 0.05755 (0.000–1.000)	0.444 ± 0.1631 (0.000–3.000)	0.3214 ± 0.1265 (0.000–2.000)
Tertiary lysosomes (n)	0.5556 ± 0.1445 (0.000–3.000)	0.2069 ± 0.07655 (0.000–1.000)	0.1852 ± 0.09302 (0.000–2.000)	0.3571 ± 0.1282 (0.000–2.000)
All lysosomes (n)	0.6296 ± 0.1614 (0.000–3.000)	0.3448 ± 0.08983 (0.000–1.000)	0.7937 ± 0.2123 (0.000–4.000)	0.7500 ± 0.1677 (0.000–3.000)
Lipid bodies (n)	1.074 ± 0.2383 (0.000–4.000)	0.7586 ± 0.2748 (0.000–7.000)	0.7037 ± 0.3493 (0.000–9.000)	0.8929 ± 0.3386 (0.000–8.000)
Lipofuscin granules (n)	0.8148 ± 0.1927 (0.000–3.000)	0.6897 ± 0.1929 (0.000–3.000)	1.000 ± 0.3849 (0.000–7.000)	0.9286 ± 0.2116 (0.000–3.000)
Partially digested endosomes (n)	0.5185 ± 0.1449 (0.000–2.000)	0.9310 ± 0.2216 (0.000–4.000)	1.444 ± 0.3261 (0.000–5.000)	1.250 ± 0.3155 (0.000–6.000)
Fully digested endosomes (n)	0.3333 ± 0.1688 (0.000–4.000)	0.7931 ± 0.1674 (0.000–3.000)	0.7407 ± 0.2480 (0.000–4.000)	0.9643 ± 0.2976 (0.000–6.000)
All endosomes (n) ~	0.7407 ± 0.2174 (0.000–4.000)	1.759 ± 0.2836 (0.000–5.000)	2.185 ± 0.4685 (0.000–9.000)	2.214 ± 0.4780 (0.000–8.000)
Autophagosomes (n)	0.2222 ± 0.09745 (0.000–2.000)	0.06897 ± 0.04789 (0.000–1.000)	0.2222 ± 0.1111 (0.000–2.000)	0.3571 ± 0.1282 (0.000–3.000)
Fibrillar materials (n)	0.000 ± 0.000 (0.000–1.000)	0.06897 ± 0.04789 (0.000–1.000)	0.1111 ± 0.06163 (0.000–1.000)	0.07143 ± 0.04956 (0.000–1.000)
Altered mitochondria (n) ~ ~ ~ ~	0.3704 ± 0.1087 (0.000–2.000)	1.207 ± 0.1815% (0.000–3.000)	1.889 ± 0.4347&& (0.000–10.00)	3.464 ± 0.6289!!!! (0.000–11.00)
Elongated mitochondria (n)	0.3333 ± 0.1194 (0.000–2.000)	0.4138 ± 0.1267 (0.000–2.000)	0.4074 ± 0.1710 (0.000–3.000)	0.6071 ± 0.2376 (0.000–6.000)
Dilated ER/Golgi apparatus (n)~~~	0.2222 ± 0.1111 (0.000–2.000)	0.5862 ± 0.1955 (0.000–5.000)	1.593 ± 0.3968&& (0.000–7.000)	2.071 ± 0.4740!! (0.000–9.000)

*CTM* control typical microglia, *FTM* typical microglia far from Aβ plaque and dystrophic neurites, *NTM* typical microglia near Aβ plaque and dystrophic neurite, *NDM* dark micorglia near Aβ plaque and dystrophic neurite, *n* number, *a.u.* arbritary unit, *ER* endoplasmic reticulum

*p*-values of statistically significant tests are highlighted with various symbols (*,#,&,!,%). Data reported is shown as number per cell. Data shown are expressed as means ± SEM along with the minimum and maximum range of values obtained. ~ p < 0.05, ~  ~ p < 0.01, ~  ~  ~  ~ p < 0.001 using a Kruskal–Wallis test with a post hoc Dunn’s multiple comparisons test. ~ p-value summary, * NDM vs NTM, # NDM vs FTM, ! NDM vs CTM, & NTM vs CTM, % FTM vs CTM. Statistical tests were performed on n = 9–12 microglia per animal with N = 3 mice/group, for a total of 111 microglial cell bodies analyzed (effect size of 0.4, power of 0.95 determined by G*Power Software V3.1)

**Table 2:** Relative ultrastructural analysis of dark microglia near Aβ plaques and dystrophic neurites compared to typical microglia near vs far Aβ plaques and dystrophic neurites in the ventral hippocampus CA1 *stratum lacunosum-moleculare* of aged-matched APP-PS1 vs C57BL/6J male mice

	C57BL/6J	APP-PS1		
	CTM Mean ± SEM	FTM Mean ± SEM	NTM Mean ± SEM	NDM Mean ± SEM
Associations with myelinated axons (%) ~ ~ ~	33.33 ± 9.245	37.93 ± 9.170	11.11 ± 6.163	0.000 ± 0.000##!
Extracellular space (%) ~	25.93 ± 8.594	51.72 ± 9.443	14.81 ± 6.967	21.43 ± 7.897*
Extracellular digestion (%)	25.93 ± 8.594	17.24 ± 7.139	14.81 ± 6.967	7.143 ± 4.956
Primary lysosomes (%)	0.000 ± 0.000	3.448 ± 3.448	7.407 ± 5.136	7.143 ± 4.956
Secondary lysosomes (%)	3.704 ± 3.704	10.34 ± 5.755	18.59 ± 7.611	21.43 ± 7.897
Tertiary lysosomes (%)	44.44 ± 9.745	20.69 ± 7.655	14.81 ± 6.967	25.00 ± 8.333
All lysosomes (%)	44.44 ± 9.745	34.48 ± 8.983	37.04 ± 9.471	50.00 ± 9.623
Lipid bodies (%)	55.56 ± 9.745	34.48 ± 8.983	29.63 ± 8.955	32.14 ± 8.988
Lipofuscin granules (%)	44.44 ± 9.745	37.93 ± 9.170	29.63 ± 8.955	50.00 ± 9.623
Partially digested endosomes (%)	37.04 ± 9.471	48.28 ± 9.443	55.56 ± 9.745	57.14 ± 9.524
Fully digested endosomes (%)	18.52 ± 7.618	51.72 ± 9.443	33.33 ± 9.245	50.00 ± 9.623
All endosomes (%)	40.74 ± 9.636	72.41 ± 8.447	59.26 ± 9.636	67.86 ± 8.988
Autophagosomes (%)	18.52 ± 7.618	6.897 ± 4.789	14.81 ± 6.967	28.57 ± 8.694
Altered mitochondria (%) ~ ~	33.33 ± 9.245	72.41 ± 8.447	70.37 ± 8.955	78.57 ± 7.897*
Elongated mitochondria (%)	25.93 ± 8.594	31.03 ± 8.743	22.22 ± 8.153	32.14 ± 8.988
Dilated ER/Golgi apparatus (%) ~ ~	14.81 ± 6.967	37.93 ± 9.170	55.56 ± 9.745	60.71 ± 9.399*
Glycogen granules (%) ~ ~ ~ ~	0.000 ± 0.000	3.448 ± 3.448	29.63 ± 8.955	60.71 ± 9.399*
Associations with vasculature (%) ~ ~	33.33 ± 9.245	6.897 ± 4.789	3.704 ± 3.704	3.571 ± 3.571*

*CTM* control typical microglia, *FTM* typical microglia far from amyloid beta plaque and dystrophic neurite, *NTM* typical microglia near amyloid beta plaque and dystrophic neurite, *NDM* dark micorglia near amyloid beta plaque and dystrophic neurite, % percentage, *a.u.* arbritary unit, *ER* endoplasmic reticulum

*p*-values of statistically significant tests are highlighted with various symbols (*,#,&,!,%). Data reported is shown as % of cells positive for at least one of the elements analyzed for each category. Data shown are expressed as means ± SEM. ~ p < 0.05, ~  ~ p < 0.01, ~  ~  ~  ~ p < 0.001 using a Kruskal–Wallis test with a post hoc Dunn’s multiple comparisons test. ~ p-value summary, * NDM vs NTM, # NDM vs FTM, ! NDM vs CTM, & NTM vs CTM, % FTM vs CTM. Statistical tests were performed on n = 9–12 microglia per animal with N = 3 mice/group, for a total of 111 microglial cell bodies analyzed (effect size of 0.4, power of 0.95 determined by G*Power Software V3.1)

**Fig. 4:**
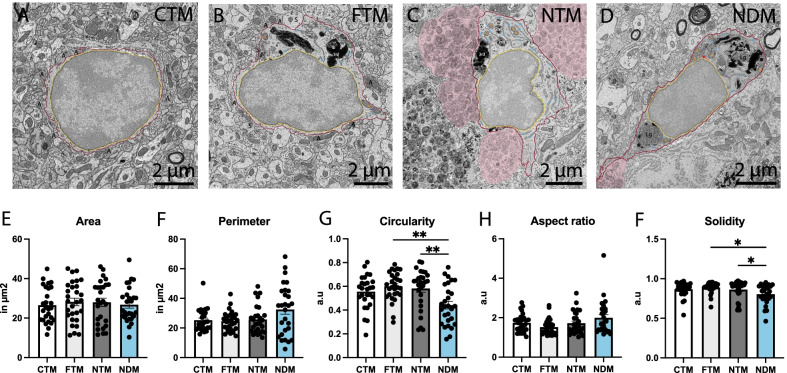
Shape descriptors of dark vs typical microglia. Representative 5 nm resolution scanning electron microscopy images captured in the ventral hippocampus CA1 *stratum lacunosum-moleculare* of 20-month-old APP-PS1 and C57BL/6J male mice. **A** typical microglia (TM) in C57BL/6J mice, **B** TM far from Aβ plaque, (**C**) TM near Aβ plaques and dystrophic neurites, (**D**) dark microglia (DM) near Aβ plaques and dystrophic neurites in APP-PS1 mice. **E**–**I** Graphs representing the shape descriptors of microglia: (**E**) area, (**F**) perimeter, (**G**) circularity, (**H**) aspect ratio and (**I**) solidity. Data shown are expressed as means ± S.E.M. *p < 0.05, **p < 0.01, using a Kruskal–Wallis test with a Dunn’s multiple comparisons post hoc test. Statistical tests were performed on n = 9–12 microglia per animal with N = 3 mice/group, for a total of 111 microglial cell bodies analyzed. Red outline = plasma membrane, yellow outline = nuclear membrane. A = axon terminals, S = dendritic spines, orange asterisk = mitochondria, green asterisk = altered mitochondria, blue asterisk = endoplasmic reticulum, red arrow = Golgi apparatus, lb = lipid body, 3rd = tertiary lysosome, 2nd = secondary lysosome, Lg = lipofuscin granules, pink pseudo-coloring = dystrophic neurites

In terms of interactions with axonal myelination, which was shown to be affected in AD [100], we found that dark microglia located near Aβ plaques and dystrophic neurites contacted less myelinated axons compared to typical microglia far from Aβ plaques and dystrophic neurites and typical microglia in control C57BL/6J mice (NDM 0.000 ± 0.000 contact per microglia vs FTM 0.6207 ± 0.1818 contact per microglia, *p* = 0.0035; NDM 0.000 ± 0.000 contact per microglia vs CTM 0.5556 ± 0.1716 contact per microglia, *p* = 0.0152). Similarly, dark microglia located near Aβ plaques and dystrophic neurites juxtaposing at least one myelinated axon were significantly reduced in number compared to typical microglia far from the two AD hallmarks and to C57BL/6J typical microglia (NDM 0.000 ± 0.000% vs CTM 33.33 ± 9.245%, *p* = 0.0144; NDM 0.000 ± 0.000% vs FTM 37.93 ± 9.170%, *p* = 0.0026) (Fig. 3H, I). However, these interactions were similar between dark and typical microglia located near Aβ plaques and dystrophic neurites, suggesting that this difference in contact with myelinated axons is not due to the proximity to AD pathology hallmarks. Moreover, we confirmed that these changes in parenchymal interactions were not associated with a change in the size of these cells (i.e., increased perimeter or area of the cell body’s nucleoplasm and cytoplasm). Indeed, we did not observe any differences in the area, perimeter, and aspect ratio of the dark microglia located near Aβ plaques and dystrophic neurites compared to all the typical microglia groups (Fig. 4E, F). Nevertheless, we found a significant reduction in the circularity (NDM 0.4417 ± 0.03181 a.u. vs NTM 0.5820 ± 0.03226 a.u., *p* = 0.0082; NDM 0.4417 ± 0.03181 a.u. vs FTM 0.5993 ± 0.02212 a.u., *p* = 0.0043) and solidity (NDM 0.8038 ± 0.02264 a.u. vs NTM 0.8620 ± 0.02245 a.u., *p* = 0.0324; NDM 0.8038 ± 0.02264 a.u. vs FTM 0.8891 ± 0.01269 a.u., *p* = 0.0164) of the dark microglia observed near Aβ plaques and dystrophic neurites compared to typical microglia both localized near and far from Aβ plaques and dystrophic neurites in APP-PS1 mice. This finding suggests an increased irregularity of their cell body shape (Fig. 4), previously associated with membrane ruffling [101] and the presence of minute pseudopodia [102].

While our previous studies identified dark microglia near the vasculature (e.g., mouse models of chronic stress, CX3CR1 knockout, middle-aged C57BL/6J and APP/PS1 mice), we rarely observed this association in the aged APP-PS1 mice. This change may be attributed to the genotype rather than the cellular state, as all microglia (both typical and dark) observed in the APP-PS1 mice interacted significantly less with the vasculature compared to C57BL/6J typical microglia, regardless of their distance to Aβ plaques and dystrophic neurites (NDM 3.571 ± 3.571% vs CTM 33.33 ± 9.245%, *p* = 0.0038; NTM 3.704 ± 3.704% vs CTM 33.33 ± 9.245%, *p* = 0.0045; FTM 6.897 ± 4.789% vs CTM 33.33 ± 9.245%, *p* = 0.0133) (Fig. 3G). This drastic difference in dark microglial vascular interactions found in the aged APP-PS1 mice compared to the controls could be associated with a previously observed reduced volume of cerebral vasculature near Aβ plaques, a phenomenon suggested to be caused by non-productive angiogenesis resulting in vascular scars near Aβ plaques [103].

Overall, in parallel with an increased interaction with dystrophic neurites, dark microglia also interacted less with non-dystrophic axon terminals and myelinated axons, as well as blood vessels, highlighting their preferential interactions with dystrophic neurites during aging and Aβ pathology.

### Dark microglia possess more ultrastructural markers of cellular stress compared to typical microglia in aged C57BL6/J mice

**Fig. 5:**
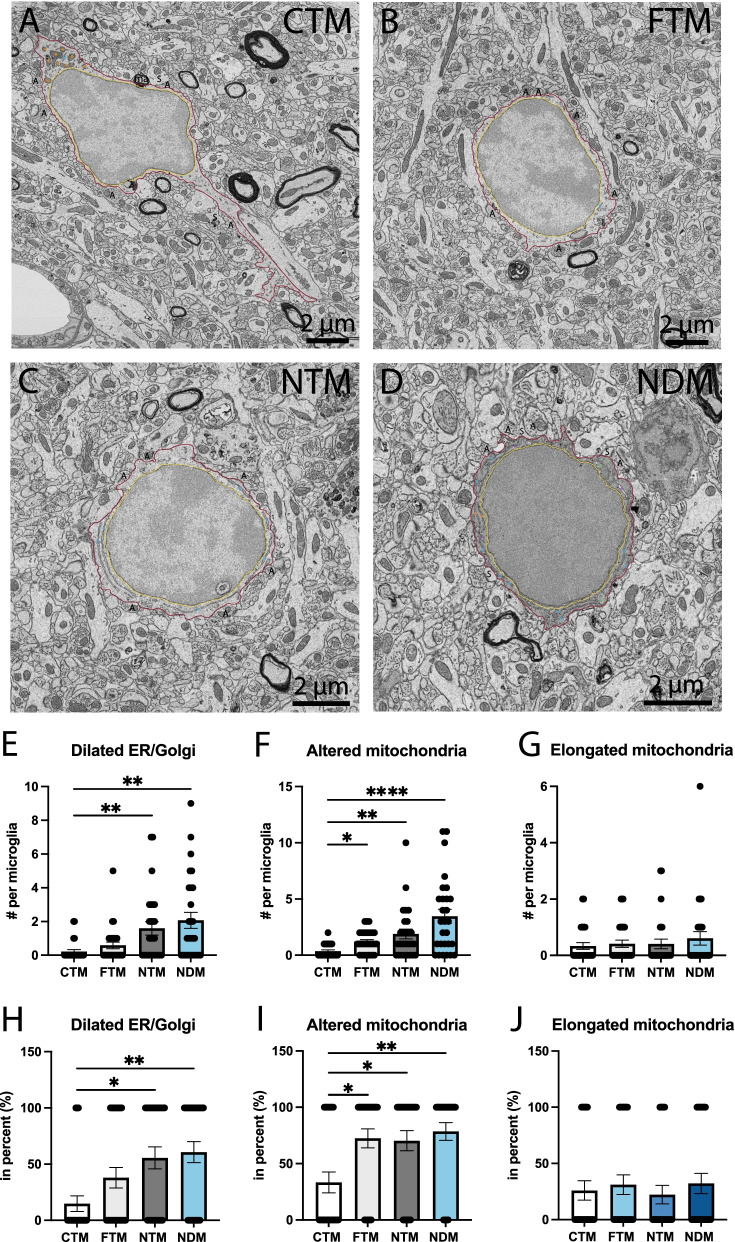
Ultrastructural signs of cellular stress in dark vs typical microglia. Representative 5 nm resolution scanning electron microscopy images taken in the ventral hippocampus CA1 *stratum lacunosum-moleculare* of 20-month-old APP-PS1 and C57BL/6J male mice. **A** Typical microglia (TM) in C57BL/6J mice without visible ultrastructural signs of cellular stress, **B** TM far from a plaque with altered mitochondria (green asterisk) (**C**) TM near a plaque with healthy endoplasmic reticulum cisternae (blue asterisk), (**D**) dark microglia (DM) near a plaque with altered mitochondria (green asterisk), healthy and dilated endoplasmic reticulum cisternae (blue and purple asterisks respectively) in APP-PS1 mice. Graphs representing the number and proportion of microglial cells with dilated endoplasmic reticulum and/or Golgi apparatus cisternae (**E**, **H**), altered mitochondria (**F**, **I**) and elongated mitochondria (**G**, **J**) are presented. Data shown are expressed as means ± S.E.M. * p < 0.05, ** p < 0.01, **** p < 0.001 using a Kruskal–Wallis test with a Dunn’s multiple comparisons post hoc test. Statistical tests were performed on n = 9–12 microglia per animal with N = 3 mice/group, for a total of 111 microglial cell bodies analyzed. Red outline = plasma membrane, yellow outline = nuclear membrane, ma = myelinated axons, A = axon terminals, S = dendritic spines, orange asterisk = mitochondria, green asterisk = altered mitochondria, blue asterisk = endoplasmic reticulum, purple asterisk = dilated endoplasmic reticulum

Considering that dark microglia were previously described as phagocytic cells with several ultrastructural markers of cellular stress (e.g., altered mitochondria, dilated ER, electron-dense cyto- and nucleoplasm) [64, 65, 76], we next investigated the cellular content of these cells in the context of aging and Aβ pathology. Although we noticed a main effect due to the genotype (C57BL/6J vs APP-PS1) (*p* = 0.0331) at 20 months of age, we did not find significant differences in the number of phagosomes (partially and fully digested) and number of cells containing at least one phagosome between all the microglial groups examined (C57BL/6J typical microglia, APP-PS1 typical microglia far vs near Aβ plaques and dystrophic neurites, APP-PS1 dark microglia near Aβ plaques and dystrophic neurites). All the APP-PS1 microglia, both typical and dark, showed a significant increase in their number of altered mitochondria compared to C57BL/6J typical microglia (NDM 3.464 ± 0.6289 per microglia vs CTM 0.3704 ± 0.1087 per microglia, *p* < 0.0001; NTM 1.889 ± 0.4347 per microglia vs CTM 0.3704 ± 0.1087 per microglia, *p* = 0.0072; FTM 1.207 ± 0.1815 per microglia vs CTM 0.3704 ± 0.1087 per microglia, *p* = 0.0362). Additionally, more APP-PS1 dark and typical microglia contained ultrastructurally-altered mitochondria compared to C57BL/6J typical microglia (NDM 78.57 ± 7.897% vs CTM 33.33 ± 9.245%, *p* = 0.0030; NTM 70.37 ± 8.955% vs CTM 33.33 ± 9.245%, *p* = 0.0287; FTM 72.41 ± 8.447% vs CTM 33.33 ± 9.245%, *p* = 0.0147) (Fig. 5F, I).

In addition, dark and typical microglia near Aβ plaques and dystrophic neurites showed a significant increase in the number of dilated ER compared to the C57BL/6J typical microglia (NDM 2.071 ± 0.4740 per microglia vs CTM 0.2222 ± 0.1111 per microglia, *p* = 0.0010; NTM 1.593 ± 0.3968 per microglia vs CTM 0.2222 ± 0.1111 per microglia, *p* = 0.0081). Similarly, we found an increased number of microglia near Aβ plaques and dystrophic neurites containing at least one dilated ER cisternae compared to C57BL/6J typical microglia (NDM 60.71 ± 9.399% vs CTM 14.81 ± 6.967%, *p* = 0.0036; NTM 55.56 ± 9.745% vs CTM 14.81 ± 6.967%, *p* = 0.0154). Since both dark and typical microglia near Aβ plaques and dystrophic neurites contained more dilated ER cisternae, the distribution of these cells (i.e., association with Aβ plaques and dystrophic neurites) was likely underlying this increased prevalence. While dark microglia near Aβ plaques and dystrophic neurites possess numerous markers of cellular stress, there were no quantitative differences between typical microglia located near vs far from Aβ plaques and dystrophic neurites. In short, both dark and typical microglia in APP-PS1 mice can possess signs of cellular stress, denoting the impact of AD pathology on the two microglial states.

### Dark and typical microglia located near Aβ plaques and dystrophic neurites contain glycogen granules in aged APP-PS1 mice

**Fig. 6:**
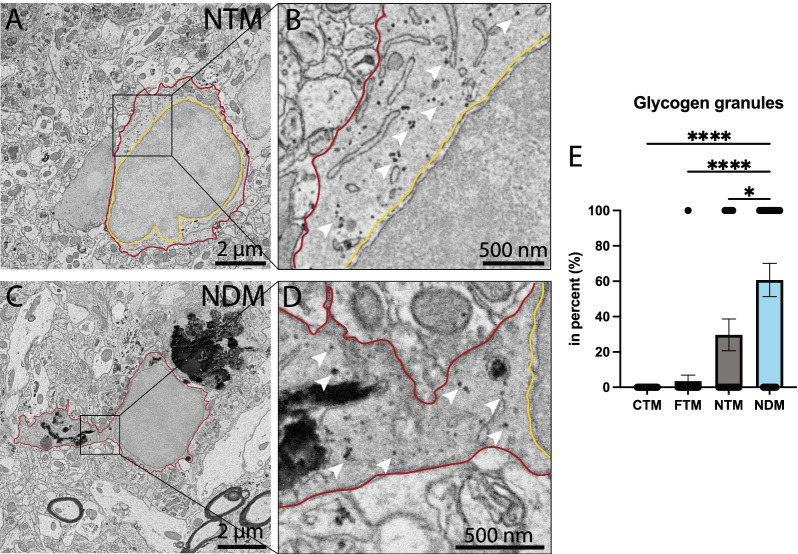
Glycogen granules in the cytoplasm of dark vs typical microglia. Representative 5 nm resolution scanning electron microscopy images captured in the ventral hippocampus CA1 *stratum lacunosum-moleculare* of 20-month-old APP-PS1 male mice. **A** Typical microglia (TM) near extracellular fibrillar Aβ (NTM) and (**C**) dark microglia (DM) near Aβ (NDM), both presenting glycogen granules in their cytoplasm (white arrow). **B**–**D** represent the insets of **A** and **C**, respectively. **E** Graph representing the proportion of microglia positive for glycogen granules. Data shown are expressed as means ± S.E.M. *p < 0.05, ****p < 0.001 using a Kruskal–Wallis test with a post hoc Dunn’s multiple comparisons test. Statistical tests were performed on n = 9–12 microglia per animal with N = 3 mice/group, for a total of 111 microglial cell bodies analyzed. Red outline = plasma membrane, yellow outline = nuclear membrane, while arrow = glycogen granules

While normally absent in microglia or observed at very low levels [72, 104], we found ultrastructural evidence of glycogen granules in the cytoplasm of dark and typical microglia located near Aβ plaques and dystrophic neurites. Sequestered glycogen, along with the predominant metabolic shift to glucose metabolism, was previously associated in myeloid cells with a pro-inflammatory response [61]. The percentage of dark microglia containing glycogen granules was significantly higher compared to typical microglia located far from Aβ plaques and dystrophic neurites and typical microglia in C57BL/6J control mice (NDM 60.71 ± 9.399% vs CTM 0.000 ± 0.000%, *p* < 0.0001; NDM 60.71 ± 9.399% vs FTM 3.448 ± 3.488%, *p* < 0.0001). These glycogen granules were distributed throughout the cytoplasm of microglial cells located near Aβ plaques and dystrophic neurites (Fig. 6). An accumulation of glycogen granules was observed in ~ 60% of all dark microglia observed in the *stratum lacunosum-moleculare* of APP-PS1 mice, while ~ 30% of typical microglia near Aβ plaques and dystrophic neurites contained a minimum of five glycogen granules within their cytoplasm (NDM 60.71 ± 9.399% vs NTM 29.63 ± 8.955%, *p* = 0.0405). We observed a very low percentage (~ 3%) of typical microglia located far from Aβ plaques and dystrophic neurites containing cytoplasmic glycogen granules, while the latter were not found in any typical microglia in the C57BL/6J mice (Fig. 6). These findings indicate that glycogen granule accumulation is largely associated with hallmarks of AD pathology and neurodegeneration. This ultrastructural finding suggests that microglia located near Aβ plaques and dystrophic neurites possess a feature traditionally associated with glycolysis, which is further exacerbated by the cellular state (dark vs typical microglia).

### Dark microglia are found in human post-mortem brain samples from middle-aged and aged individuals

**Fig. 7:**
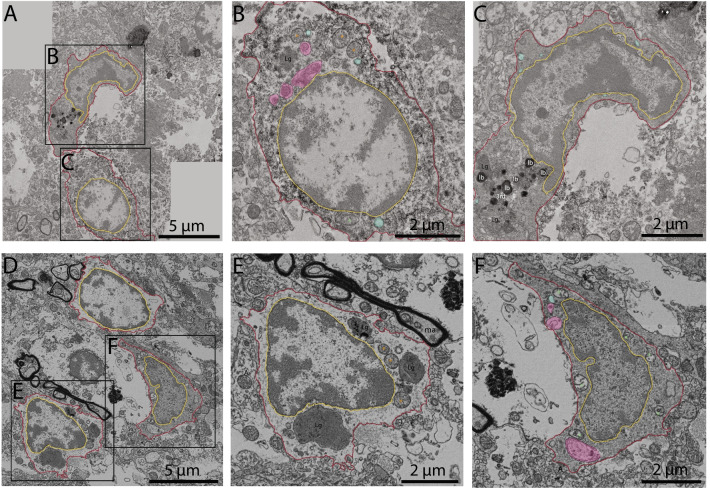
Dark vs typical microglia in aging human post-mortem brain samples. Representative 5 nm resolution scanning electron microscopy images in human post-mortem hippocampal head (post-mortem delay of 18 h) of (**A**) a 45-year old man and (**B**) a 81-year old woman. **A** Typical microglia (TM) positive for the myeloid cell marker Iba1 with healthy and altered mitochondria located next to a dark microglia (DM). The insets provide a zoom in view of the (**B**) TM and (**C**) DM. **D** TM with lipofuscin (labeled Lg) and mitochondria (orange asterisk) next to a DM displaying altered mitochondria (green asterisk), empty phagosomes (pseudocolored in cyan) and phagosomes with cellular contents (pseudocolored in pink). The insets provide a higher magnification of the (**E**) TM and (**F**) DM. Red outline = plasma membrane, yellow outline = nuclear membrane, ma = myelinated axons, A = axon terminals, orange asterisk = mitochondria, green asterisk = altered mitochondria, lb = lipid body, 3^rd^ = tertiary lysosome, Lg = lipofuscin granules, pink pseudo-coloring = partially digested phagosome, cyan pseudo-coloring = fully digested phagosome

While dark microglia were previously reported at adulthood in several mouse models of pathology (e.g., prenatal and maternal immune activation, middle-aged APP-PS1, chronic stress, CX3CR1 knockout, R6/2 model of Huntington’s disease; [64, 78, 94, 105, 106]), their presence in the human brain had yet to be reported. We observed dark cells possessing ultrastructural features of dark microglia in the hippocampal head of post-mortem brain samples from a 49-year-old man and in the parahippocampal gyrus from an 81-year-old woman (both with a post-mortem interval of 18 h). To the authors’ best knowledge, this case report is the first instance of dark microglia shown in the CNS of human post-mortem samples. Further studies using a sample size necessary to reach statistical power would be required to quantitatively investigate the intracellular contents and intercellular relationships of human dark microglia. Similar to dark microglia described in mice, dark microglia in these human brains were characterized by their electron-dense cyto- and nucleoplasm, however, still possessing in this case a relatively well-defined eu- and heterochromatin pattern. In addition, similar to the dark microglia described in mice, numerous altered mitochondria, identified by the electron-lucent space within the organelle and deterioration of the outer and inner membranes, were observed in the human dark microglia (Fig. 7D–F). Several lipofuscin granules, which are considered a marker of cellular senescence [107], were observed in the cytoplasm of the human dark microglia (Fig. 7A–C). Moreover, dark microglia were seen making direct contacts with axon terminals. We further did observe a positive Iba1 immunostaining on the human dark microglia, compared to the low to no immunoreactivity to Iba1 observed in the dark microglia from mice [64], suggesting, along with the still-visible chromatin pattern, that these microglia are an intermediate dark state (Fig. 7A–C).

## Discussion

Microglial heterogeneity has become a topic of high interest with the discovery of numerous microglial states in mouse models of AD pathology, such as the DAM [62], MGnD [63] and plaque-associated microglia (PAM) [41, 44, 108–110]. The features distinguishing these states from typical microglia include an absent or reduced gene expression or immunoreactivity for microglial homeostatic markers (e.g., P2RY12, TMEM119), a unique molecular signature (e.g., *lpl*, *clec7a*, *cd9*) [34], as well as diverse proposed roles which can be beneficial or detrimental depending on the context (e.g., clearance of Aβ, propagation of tau) [12, 108]. The dark microglia, a state previously identified in adult mouse models of pathology, including AD, was characterized by its distinctive ultrastructural appearance (i.e., electron-dense cyto- and nucleoplasm, altered mitochondria, dilated ER cisternae, loss of heterochromatin pattern). Our previous work has identified the preferential location of these cells near Aβ plaques and dystrophic neurites, and their extensive interactions with synaptic elements (axon terminals, dendritic spines, both elements of a same synapse) [64, 65], suggesting a key role in the synaptic dysfunction observed in AD [1].

The current study investigated the distribution and ultrastructure of dark vs typical microglia based on their distance to Aβ plaques and dystrophic neurites in the ventral hippocampus CA1 *stratum lacunosum-moleculare*, where dark microglia were previously observed in high numbers [64, 65], within 20-month-old APP-PS1 and C57BL/6J male mice. We found that while dark microglia were barely present in aged 20-month-old C57BL/6J male mice, their density increased drastically with Aβ plaques and dystrophic neurites, reaching 43% of all the microglia found nearby Aβ plaques and dystrophic neurites in the *stratum lacunosum-moleculare* of age-matched APP-PS1 mice*.* The exact role of dark microglia in the pathogenesis of AD remains unclear. The importance of dark microglia’s interventions near Aβ plaques and dystrophic neurites is supported by the finding that more than half of all the fibrillar Aβ plaques observed had at least one dark microglia present within its micro-environment. This presence of dark microglia near Aβ plaques was likely underestimated as the ultrathin sections examined do not provide a 3D view of the plaques, therefore there were likely many additional cells located above and below the 70 nm plane imaged. Whether dark microglia are a result of their proximity to Aβ plaques and dystrophic neurites, for instance arising from typical microglia that switch their metabolism to glycolysis which results in cellular stress, remains to be investigated. Of note, we do observe several markers of cellular stress (e.g., dilated ER, altered mitochondria) in dark microglia which possess glycogen granules; all signs pointing towards a shift in their metabolism to the less effective glycolysis. In addition, the dark microglia preferentially interacted with dystrophic neurites compared to typical microglia observed near Aβ plaques and dystrophic neurites, a feature that we previously reported without performing yet quantification [64]. The internalization of dystrophic neurites was previously attributed to reactive astrocytes in the hippocampus of 6 and 12-month-old APP-PS1 male mice [111]. While we did not observe dark microglia internalizing dystrophic elements, a previous study has identified dystrophic neurites inside of dark microglial cells [64]. Therefore, it remains inconclusive how dark microglia interact with dystrophic neurites over the course of AD pathology and whether this state results from and/or contributes to the appearance of dystrophic neurites. In addition to our findings in regards to dystrophic neurites and dark microglia, in the current study we showed an increased abundance of ultrastructural markers of cellular stress (altered mitochondria, dilated ER) in the microglia located near Aβ plaques and dystrophic neurites, together with altered parenchymal interactions (reduced contacts with the vasculature) compared to typical microglia in age-matched C57BL/6J mice. This is in line with our previous findings identifying dilated ER in microglia located near Aβ plaques and dystrophic neurites in the ventral hippocampus CA1 of 14-month-old APP-PS1 male mice [65].

A distinct feature of plaque-associated microglia is their metabolic shift from a primarily oxidative phosphorylation to glycolysis, known in cancer cells as the Warburg effect [54–57]. This shift in plaque-associated microglia was shown to result from the presence of Aβ [54, 55] and elevated levels of iron [55, 57], an electron-dense metal that can trigger the production of ROS, chemicals known to be associated with cellular stress (e.g., dysfunctional mitochondria) [112]. Iron accumulation in plaque-associated microglia was reported both in mouse models of AD pathology and human post-mortem brain samples [54, 57, 57, 113]. The accumulation of iron within microglial states will be interesting to investigate in future studies, as it could account for the electron-dense nature of the dark microglia, the presence of several signs of cellular stress (that can be attributed to oxidative stress, and therefore the presence of ROS) as well as the occurrence of glycogen granules. Glycogen, which is broken down during glycolysis, can be used as an energy source by CNS cells such as astrocytes, and has been shown to be crucial for memory and learning processes [91, 104]. Glycogen accumulation in myeloid cells was observed in 20–24-month old C57BL/6J mice, which was attributed to the prostaglandin E_2_ (PGE2)-protaglandin E2 receptor 2 (EP2) signaling pathway [61]. Inhibition of this pathway in the periphery restored long-term potentiation to youthful levels in the hippocampus CA1, suggesting that this metabolic switch toward glycolysis has detrimental effects on cognitive functions during aging in mice [61]. Moreover, microglia observed near Aβ deposition in 19–20 month-old APP-PS1 male and female mice were found to accumulate iron while expressing 6-phosphofructo-2-kinase/fructose-2,6-biphosphate 3 (PFKFB-3), a regulator of glycolysis [55]. In our study, the accumulation of glycogen granules was restricted to microglia (both typical and dark states, but especially dark ones) found nearby Aβ plaques, suggesting that fibrillar Aβ could also be a key player in driving this metabolic shift. To the authors’ knowledge, this is the first ultrastructural evidence of glycogen granule accumulation in microglia. Glycogen granules were not observed in the human post-mortem brain samples that we examined, probably due to the rapid depletion of glycogen over post-mortem time [104], which prevented their investigation in human dark microglia.

In addition to our quantitative analysis in mice, we report for the first time the presence of dark microglia as a case report in post-mortem human brain samples, among the parahippocampal gyrus and hippocampal head of a 45-year-old man and an 81-year-old woman, respectively. Microglia appearing ultrastructurally more electron-dense than typical microglia were previously shown nearby oligodendrocytes in prefrontal white matter post-mortem samples from a patient with schizophrenia [114]. However, these microglial cells were described as “dystrophic”, while ultrastructural signs of oxidative stress, a feature of dark microglia, were not investigated in this study. To the best of our knowledge, this is the first time that dark microglia (i.e., microglia with a dense cyto- and- nucleoplasm and ultrastructural signs of oxidative stress) are reported in human post-mortem brain samples. The conservation of key features associated with dark microglia across mouse and human species, including an electron dense cyto- and nucleoplasm, altered mitochondria and dilated ER cisternae, along with several partially to fully-digested phagosomes, emphasizes the need to elucidate the mechanisms behind the appearance of this state and its role in AD.

## Conclusion

Our ultrastructural investigation of microglial states in AD pathology revealed that dark microglia are preferentially distributed near Aβ and dystrophic neurites, appearing after these hallmarks emerge in the ventral hippocampus CA1 *stratum lacunosum-moleculare* of 20-month-old APP-PS1 and C57BL/6J male mice. The dark microglia interacted more with dystrophic neurites compared to typical microglia located near Aβ plaques and dystrophic neurites, and less with non-dystrophic axon terminals than typical microglia located far from Aβ plaques and dystrophic neurites. In addition, glycogen granules which are associated with a metabolic shift toward glycolysis were observed inside the cytoplasm of the typical and especially dark microglia located near Aβ plaques and dystrophic neurites, highlighting the major impact these AD hallmarks have on the metabolism and intracellular content of these cells. It will be important in future studies to determine the functional outcome of this metabolic shift in dark microglia. Lastly, the dark microglia were observed in middle-aged and aged individuals, among post-mortem samples of the parahippocampal gyrus and hippocampal head, respectively, further identifying microglial ultrastructural silimarities between mice and humans, and supporting the translational relevance of this investigation.

## Supplementary Information

**Additional file 1: Fig S1 **Presence of dark vs typical microglia in 8-month-old APP-PS1 mice. Representative 5 nm resolution of scanning electron microscopy images captured in the ventral hippocampus CA1 *stratum lacunosum-moleculare* of 8-month-old APP-PS1 and C57BL/6J mice. (A) typical microglia (TM) in C57BL/6J mice interacting with axon terminals (labeled A) and dendritic spines (labeled S), (B) TM far from a plaque interacting with synaptic elements, (C) TM near plaques with dilated endoplasmic reticulum cisternae (purple asterisk), juxtaposing synaptic elements and dystrophic neurites (pseudocolored in pink), (D) dark microglia (DM) near Aβ plaques with dilated endoplasmic reticulum cisternae and interacting with non-dystrophic and dystrophic synaptic elements in APP-PS1 mice. Red outline = plasma membrane, yellow outline = nuclear membrane. ma = myelinated axons, A = axon terminals, orange asterisk = mitochondria, green asterisk = altered mitochondria, blue asterisk = endoplasmic reticulum, purple asterisk = dilated endoplasmic reticulum, 2^rd^ = tertiary lysosome, pink pseudo-coloring = dystrophic neurites, purple pseudo-coloring = fibrillar Aβ.

## Data Availability

All data presented in this study are available from the corresponding author upon reasonable request.

## Abbreviations

AD: Alzheimer’s disease
AR: Aspect ratio
ATP: Adenosine triphosphate
ARM: Activated-response microglia
CNS: Central nervous system
CDM: Control dark microglia
CTM: Control typical microglia
DAM: Disease-associated microglia
DM: Dark microglia
E: Embryonic
ER: Endoplasmic reticulum
FDM: Dark microglia far from AD hallmarks
FTM: Typical microglia far from AD hallmarks
GWAS: Genome-wide association studies
Iba1: Ionized calcium binding adaptor molecule 1
LOAD: Late-onset Alzheimer’s disease
MgND: Microglial neurodegenerative phenotype
NDM: Dark microglia near AD hallmarks
NFT: Neurofibrillary tangles
NTM: Typical microglia near AD hallmarks
PAM: Plaque-associated microglia
PB: Phosphate buffer
PBS: Phosphate-buffered saline
PFKFB-3: 6-Phosphofructo-2-Kinase/Fructose-2,6-Biphosphate 3
SEM: Scanning electron microscope
TBS: Tris-buffered saline
TM: Typical microglia

## References

[1] Spires-Jones TL, Hyman BT (2014). The intersection of amyloid beta and tau at synapses in Alzheimer’s disease. Neuron.

[2] Terry RD, Masliah E, Salmon DP, Butters N, DeTeresa R, Hill R (1991). Physical basis of cognitive alterations in Alzheimer’s disease: synapse loss is the major correlate of cognitive impairment. Ann Neurol.

[3] Fjell AM, McEvoy L, Holland D, Dale AM, Walhovd KB (2014). Alzheimer’s Disease Neuroimaging Initiative What is normal in normal aging? Effects of aging, amyloid and Alzheimer’s disease on the cerebral cortex and the hippocampus. Prog Neurobiol.

[4] Pini L, Pievani M, Bocchetta M, Altomare D, Bosco P, Cavedo E (2016). Brain atrophy in Alzheimer’s disease and aging. Ageing Res Rev.

[5] Masurkar AV (2018). Towards a circuit-level understanding of hippocampal CA1 dysfunction in Alzheimer’s disease across anatomical axes. J Alzheimers Dis Parkinsonism.

[6] Selles MC, Oliveira MM, Ferreira ST (2018). Brain inflammation connects cognitive and non-cognitive symptoms in Alzheimer’s disease. J Alzheimers Dis.

[7] Heneka MT, Carson MJ, El Khoury J, Landreth GE, Brosseron F, Feinstein DL (2015). Neuroinflammation in Alzheimer’s disease. Lancet Neurol.

[8] Cabinio M, Saresella M, Piancone F, LaRosa F, Marventano I, Guerini FR (2018). Association between hippocampal shape, neuroinflammation, and cognitive decline in Alzheimer’s disease. J Alzheimers Dis.

[9] Kinney JW, Bemiller SM, Murtishaw AS, Leisgang AM, Salazar AM, Lamb BT (2018). Inflammation as a central mechanism in Alzheimer’s disease. Alzheimers Dement (N Y).

[10] Hansen DV, Hanson JE, Sheng M (2018). Microglia in Alzheimer’s disease. J Cell Biol.

[11] Sala Frigerio C, Wolfs L, Fattorelli N, Thrupp N, Voytyuk I, Schmidt I (2019). The major risk factors for Alzheimer’s disease: age, sex, and genes modulate the microglia response to Aβ plaques. Cell Rep.

[12] Sebastian Monasor L, Müller SA, Colombo AV, Tanrioever G, König J, Roth S (2020). Fibrillar Aβ triggers microglial proteome alterations and dysfunction in Alzheimer mouse models. Elife.

[13] Lodder C, Scheyltjens I, Stancu IC, BotellaLucena P, Gutiérrez de Ravé M, Vanherle S (2021). CSF1R inhibition rescues tau pathology and neurodegeneration in an A/T/N model with combined AD pathologies, while preserving plaque associated microglia. Acta Neuropathol Commun.

[14] Vogels T, Murgoci AN, Hromádka T (2019). Intersection of pathological tau and microglia at the synapse. Acta Neuropathol Commun.

[15] Sosna J, Philipp S, Albay R, Reyes-Ruiz JM, Baglietto-Vargas D, LaFerla FM (2018). Early long-term administration of the CSF1R inhibitor PLX3397 ablates microglia and reduces accumulation of intraneuronal amyloid, neuritic plaque deposition and pre-fibrillar oligomers in 5XFAD mouse model of Alzheimer’s disease. Mol Neurodegener.

[16] Spangenberg E, Severson PL, Hohsfield LA, Crapser J, Zhang J, Burton EA (2019). Sustained microglial depletion with CSF1R inhibitor impairs parenchymal plaque development in an Alzheimer’s disease model. Nat Commun.

[17] von Saucken VE, Jay TR, Landreth GE (2020). The effect of amyloid on microglia-neuron interactions before plaque onset occurs independently of TREM2 in a mouse model of Alzheimer’s disease. Neurobiol Dis.

[18] Ginhoux F, Greter M, Leboeuf M, Nandi S, See P, Gokhan S (2010). Fate mapping analysis reveals that adult microglia derive from primitive macrophages. Science.

[19] Andjelkovic AV, Nikolic B, Pachter JS, Zecevic N (1998). Macrophages/microglial cells in human central nervous system during development: an immunohistochemical study. Brain Res.

[20] Monier A, Evrard P, Gressens P, Verney C (2006). Distribution and differentiation of microglia in the human encephalon during the first two trimesters of gestation. J Comp Neurol.

[21] Verney C, Monier A, Fallet-Bianco C, Gressens P (2010). Early microglial colonization of the human forebrain and possible involvement in periventricular white-matter injury of preterm infants. J Anat.

[22] Tremblay MÈ, Stevens B, Sierra A, Wake H, Bessis A, Nimmerjahn A (2011). The role of microglia in the healthy brain. J Neurosci.

[23] Šišková Z, Tremblay MÈ. Microglia and synapse: interactions in health and neurodegeneration. Neural Plast [Internet]. 2013 [cited 2019 Dec 13];2013. Available from: https://www.ncbi.nlm.nih.gov/pmc/articles/PMC3874338/. 10.1155/2013/425845 PMC3874338 24392228

[24] Gomez-Nicola D, Perry VH (2015). Microglial dynamics and role in the healthy and diseased brain. Neuroscientist.

[25] Ransohoff RM, Khoury JE. Microglia in Health and Disease. Cold Spring Harb Perspect Biol [Internet]. 2016 Jan [cited 2019 Jan 29];8(1). Available from: https://www.ncbi.nlm.nih.gov/pmc/articles/PMC4691795/. 10.1101/cshperspect.a020560 PMC4691795 26354893

[26] Augusto-Oliveira M, Arrifano GP, Lopes-Araújo A, Santos-Sacramento L, Takeda PY, Anthony DC, et al. What Do Microglia Really Do in Healthy Adult Brain? Cells [Internet]. 2019 Oct 22 [cited 2020 Jun 22];8(10). Available from: https://www.ncbi.nlm.nih.gov/pmc/articles/PMC6829860/. 10.3390/cells8101293 PMC6829860 31652490

[27] Davalos D, Grutzendler J, Yang G, Kim JV, Zuo Y, Jung S (2005). ATP mediates rapid microglial response to local brain injury in vivo. Nat Neurosci.

[28] Nimmerjahn A, Kirchhoff F, Helmchen F (2005). Resting microglial cells are highly dynamic surveillants of brain parenchyma in vivo. Science.

[29] Parkhurst CN, Yang G, Ninan I, Savas JN, Yates JR, Lafaille JJ (2013). Microglia promote learning-dependent synapse formation through BDNF. Cell.

[30] Weinhard L, DiBartolomei G, Bolasco G, Machado P, Schieber NL, Neniskyte U (2018). Microglia remodel synapses by presynaptic trogocytosis and spine head filopodia induction. Nat Commun.

[31] Tremblay MÈ, Majewska AK (2011). A role for microglia in synaptic plasticity?. Commun Integr Biol.

[32] Bliss TVP, Collingridge GL, Morris RGM. Synaptic plasticity in health and disease: introduction and overview. Philos Trans R Soc Lond B Biol Sci [Internet]. 2014 Jan 5 [cited 2019 Jan 29];369(1633). Available from: https://www.ncbi.nlm.nih.gov/pmc/articles/PMC3843863/. 10.1098/rstb.2013.0129 PMC3843863 24298133

[33] Hong S, Dissing-Olesen L, Stevens B (2016). New insights on the role of microglia in synaptic pruning in health and disease. Curr Opin Neurobiol.

[34] Paolicelli R, Sierra A, Stevens B, Tremblay ME, Aguzzi A, Ajami B, et al. Defining Microglial States and Nomenclature: A Roadmap to 2030 [Internet]. Rochester, NY: Social Science Research Network; 2022 Mar [cited 2022 May 4]. Report No.: 4065080. Available from: https://papers.ssrn.com/abstract=4065080.

[35] Streit WJ, Sammons NW, Kuhns AJ, Sparks DL (2004). Dystrophic microglia in the aging human brain. Glia.

[36] Streit WJ, Braak H, Xue QS, Bechmann I (2009). Dystrophic (senescent) rather than activated microglial cells are associated with tau pathology and likely precede neurodegeneration in Alzheimer’s disease. Acta Neuropathol.

[37] Streit WJ, Khoshbouei H, Bechmann I (2020). Dystrophic microglia in late-onset Alzheimer’s disease. Glia.

[38] Lopes KO, Sparks DL, Streit WJ (2008). Microglial dystrophy in the aged and Alzheimer’s disease brain is associated with ferritin immunoreactivity. Glia.

[39] Swanson MEV, Murray HC, Ryan B, Faull RLM, Dragunow M, Curtis MA (2020). Quantitative immunohistochemical analysis of myeloid cell marker expression in human cortex captures microglia heterogeneity with anatomical context. Sci Rep.

[40] Yuan P, Condello C, Keene CD, Wang Y, Bird TD, Paul SM (2016). TREM2 haplodeficiency in mice and humans impairs the microglia barrier function leading to decreased amyloid compaction and severe axonal dystrophy. Neuron.

[41] Yin Z, Raj D, Saiepour N, Van Dam D, Brouwer N, Holtman IR (2017). Immune hyperreactivity of Aβ plaque-associated microglia in Alzheimer’s disease. Neurobiol Aging.

[42] Condello C, Yuan P, Grutzendler J (2018). Microglia-mediated neuroprotection, TREM2, and Alzheimer’s disease: evidence from optical imaging. Biol Psychiatry.

[43] Hashemiaghdam A, Mroczek M (2020). Microglia heterogeneity and neurodegeneration: the emerging paradigm of the role of immunity in Alzheimer’s disease. J Neuroimmunol.

[44] Grubman A, Choo XY, Chew G, Ouyang JF, Sun G, Croft NP (2021). Transcriptional signature in microglia associated with Aβ plaque phagocytosis. Nat Commun.

[45] Michaud JP, Bellavance MA, Préfontaine P, Rivest S (2013). Real-time in vivo imaging reveals the ability of monocytes to clear vascular amyloid beta. Cell Rep.

[46] Rivera-Escalera F, Pinney JJ, Owlett L, Ahmed H, Thakar J, Olschowka JA (2019). IL-1β-driven amyloid plaque clearance is associated with an expansion of transcriptionally reprogrammed microglia. J Neuroinflam.

[47] Casali BT, MacPherson KP, Reed-Geaghan EG, Landreth GE (2020). Microglia depletion rapidly and reversibly alters amyloid pathology by modification of plaque compaction and morphologies. Neurobiol Dis.

[48] Huang Y, Happonen KE, Burrola PG, O’Connor C, Hah N, Huang L (2021). Microglia use TAM receptors to detect and engulf amyloid β plaques. Nat Immunol.

[49] Daria A, Colombo A, Llovera G, Hampel H, Willem M, Liesz A (2017). Young microglia restore amyloid plaque clearance of aged microglia. EMBO J.

[50] Koellhoffer EC, McCullough LD, Ritzel RM (2017). Old maids: aging and its impact on microglia function. Int J Mol Sci.

[51] Kaur D, Sharma V, Deshmukh R (2019). Activation of microglia and astrocytes: a roadway to neuroinflammation and Alzheimer’s disease. Inflammopharmacology.

[52] Yuan C, Aierken A, Xie Z, Li N, Zhao J, Qing H (2020). The age-related microglial transformation in Alzheimer’s disease pathogenesis. Neurobiol Aging.

[53] Edler MK, Mhatre-Winters I, Richardson JR (2021). Microglia in aging and alzheimer’s disease: a comparative species review. Cells.

[54] Baik SH, Kang S, Lee W, Choi H, Chung S, Kim JI (2019). A breakdown in metabolic reprogramming causes microglia dysfunction in Alzheimer’s disease. Cell Metab.

[55] McIntosh A, Mela V, Harty C, Minogue AM, Costello DA, Kerskens C (2019). Iron accumulation in microglia triggers a cascade of events that leads to altered metabolism and compromised function in APP/PS1 mice. Brain Pathol.

[56] Guillot-Sestier MV, Araiz AR, Mela V, Gaban AS, O’Neill E, Joshi L (2021). Microglial metabolism is a pivotal factor in sexual dimorphism in Alzheimer’s disease. Commun Biol.

[57] Holland R, McIntosh AL, Finucane OM, Mela V, Rubio-Araiz A, Timmons G (2018). Inflammatory microglia are glycolytic and iron retentive and typify the microglia in APP/PS1 mice. Brain Behav Immun.

[58] Shibuya T, Tsujimoto Y (2012). Deleterious effects of mitochondrial ROS generated by KillerRed photodynamic action in human cell lines and *C. elegans*. J Photochem Photobiol B.

[59] Guo C, Sun L, Chen X, Zhang D (2013). Oxidative stress, mitochondrial damage and neurodegenerative diseases. Neural Regen Res.

[60] Zeeshan HMA, Lee GH, Kim HR, Chae HJ (2016). Endoplasmic reticulum stress and associated ROS. Int J Mol Sci.

[61] Minhas PS, Latif-Hernandez A, McReynolds MR, Durairaj AS, Wang Q, Rubin A (2021). Restoring metabolism of myeloid cells reverses cognitive decline in ageing. Nature.

[62] Keren-Shaul H, Spinrad A, Weiner A, Matcovitch-Natan O, Dvir-Szternfeld R, Ulland TK (2017). A unique microglia type associated with restricting development of Alzheimer’s disease. Cell.

[63] Krasemann S, Madore C, Cialic R, Baufeld C, Calcagno N, ElFatimy R (2017). The TREM2-APOE pathway drives the transcriptional phenotype of dysfunctional microglia in neurodegenerative diseases. Immunity.

[64] Bisht K, Sharma KP, Lecours C, Gabriela Sánchez M, El Hajj H, Milior G (2016). Dark microglia: a new phenotype predominantly associated with pathological states. Glia.

[65] El Hajj H, Savage JC, Bisht K, Parent M, Vallières L, Rivest S (2019). Ultrastructural evidence of microglial heterogeneity in Alzheimer’s disease amyloid pathology. J Neuroinflam.

[66] Stratoulias V, Venero JL, Tremblay MÈ, Joseph B (2019). Microglial subtypes: diversity within the microglial community. EMBO J.

[67] Jankowsky JL, Zheng H (2017). Practical considerations for choosing a mouse model of Alzheimer’s disease. Mol Neurodegener.

[68] Pozueta J, Lefort R, Shelanski ML (2013). Synaptic changes in Alzheimer’s disease and its models. Neuroscience.

[69] Janota CS, Brites D, Lemere CA, Brito MA (2015). Glio-vascular changes during ageing in wild-type and Alzheimer’s disease-like APP/PS1 mice. Brain Res.

[70] Jackson SJ, Andrews N, Ball D, Bellantuono I, Gray J, Hachoumi L (2017). Does age matter? The impact of rodent age on study outcomes. Lab Anim.

[71] Koizumi J (1974). Glycogen in the central nervous system. Prog Histochem Cytochem.

[72] Hirase H, Akther S, Wang X, Oe Y (2019). Glycogen distribution in mouse hippocampus. J Neurosci Res.

[73] Borchelt DR, Ratovitski T, van Lare J, Lee MK, Gonzales V, Jenkins NA (1997). Accelerated amyloid deposition in the brains of transgenic mice coexpressing mutant presenilin 1 and amyloid precursor proteins. Neuron.

[74] Mielke MM (2018). Sex and gender differences in Alzheimer’s disease dementia. Psychiatr Times.

[75] Bisht K, El Hajj H, Savage JC, Sánchez MG, Tremblay MÈ. Correlative light and electron microscopy to study microglial interactions with β-amyloid plaques. J Vis Exp. 2016;(112). 10.3791/54060 PMC4927759 27286292

[76] St-Pierre MK, Bordeleau M, Tremblay MÈ (2019). Visualizing dark microglia. Methods Mol Biol.

[77] St-Pierre MK, Carrier M, Lau V, Tremblay MÈ. Investigating microglial ultrastructural alterations and intimate relationships with neuronal stress, dystrophy, and degeneration in mouse models of Alzheimer’s disease. Methods Mol Biol. 2022;2515:29-58. doi:10.1007/978-1-0716-2409-8_3 10.1007/978-1-0716-2409-8_3 35776344

[78] St-Pierre MK, Šimončičová E, Bögi E, Tremblay MÈ. Shedding Light on the Dark Side of the Microglia. ASN Neuro [Internet]. 2020 May 22 [cited 2020 Jun 25];12. Available from: https://www.ncbi.nlm.nih.gov/pmc/articles/PMC7249604/. 10.1177/1759091420925335 PMC7249604 32443939

[79] Bordeleau M, Lacabanne C, Fernández de Cossío L, Vernoux N, Savage JC, González-Ibáñez F (2020). Microglial and peripheral immune priming is partially sexually dimorphic in adolescent mouse offspring exposed to maternal high-fat diet. J Neuroinflam.

[80] Serdar CC, Cihan M, Yücel D, Serdar MA (2021). Sample size, power and effect size revisited: simplified and practical approaches in pre-clinical, clinical and laboratory studies. Biochem Med (Zagreb).

[81] Nahirney PC, Tremblay ME. Brain ultrastructure: putting the pieces together. Front Cell Dev Biol. 2021;9. 10.3389/fcell.2021.629503/full. 10.3389/fcell.2021.629503 PMC7930431 33681208

[82] Lecours C, St-Pierre MK, Picard K, Bordeleau M, Bourque M, Awogbindin IO (2020). Levodopa partially rescues microglial numerical, morphological, and phagolysosomal alterations in a monkey model of Parkinson’s disease. Brain Behav Immun.

[83] Peters A, Palay SL, Webster de HF (1991). The fine structure of the nervous system: neurons and their supporting cells.

[84] Frackowiak J, Wisniewski HM, Wegiel J, Merz GS, Iqbal K, Wang KC (1992). Ultrastructure of the microglia that phagocytose amyloid and the microglia that produce beta-amyloid fibrils. Acta Neuropathol.

[85] Stalder M, Phinney A, Probst A, Sommer B, Staufenbiel M, Jucker M (1999). Association of microglia with amyloid plaques in brains of APP23 transgenic mice. Am J Pathol.

[86] Bordeleau M, Fernández de Cossío L, Lacabanne C, Savage JC, Vernoux N, Chakravarty M (2021). Maternal high-fat diet modifies myelin organization, microglial interactions, and results in social memory and sensorimotor gating deficits in adolescent mouse offspring. Brain Behav Immun Health..

[87] Tremblay MÈ, Zettel ML, Ison JR, Allen PD, Majewska AK (2012). Effects of aging and sensory loss on glial cells in mouse visual and auditory cortices. Glia.

[88] Tremblay MÈ, Majewska AK (2019). Ultrastructural analyses of microglial interactions with synapses. Methods Mol Biol.

[89] Mondo E, Becker SC, Kautzman AG, Schifferer M, Baer CE, Chen J (2020). A developmental analysis of juxtavascular microglia dynamics and interactions with the vasculature. J Neurosci.

[90] Hui CW, St-Pierre MK, Detuncq J, Aumailley L, Dubois MJ, Couture V (2018). Nonfunctional mutant Wrn protein leads to neurological deficits, neuronal stress, microglial alteration, and immune imbalance in a mouse model of Werner syndrome. Brain Behav Immun.

[91] Prats C, Graham TE, Shearer J (2018). The dynamic life of the glycogen granule. J Biol Chem.

[92] Hart ML, Lauer JC, Selig M, Hanak M, Walters B, Rolauffs B (2018). Shaping the cell and the future: recent advancements in biophysical aspects relevant to regenerative medicine. J Funct Morphol Kinesiol.

[93] Leyh J, Paeschke S, Mages B, Michalski D, Nowicki M, Bechmann I (2021). Classification of microglial morphological phenotypes using machine learning. Front Cell Neurosci.

[94] Savage JC, St-Pierre MK, Carrier M, El Hajj H, Novak SW, Sanchez MG, et al. Microglial physiological properties and interactions with synapses are altered at presymptomatic stages in a mouse model of Huntington’s disease pathology. J Neuroinflam. [Internet]. 2020 Apr 2 [cited 2020 Apr 13];17. Available from: https://www.ncbi.nlm.nih.gov/pmc/articles/PMC7118932/. 10.1186/s12974-020-01782-9 PMC7118932 32241286

[95] Yasumoto Y, Stoiljkovic M, Kim JD, Sestan-Pesa M, Gao XB, Diano S (2021). Ucp2-dependent microglia-neuronal coupling controls ventral hippocampal circuit function and anxiety-like behavior. Mol Psychiatry.

[96] Hou Y, Dan X, Babbar M, Wei Y, Hasselbalch SG, Croteau DL (2019). Ageing as a risk factor for neurodegenerative disease. Nat Rev Neurol.

[97] Šišková Z, Justus D, Kaneko H, Friedrichs D, Henneberg N, Beutel T (2014). Dendritic structural degeneration is functionally linked to cellular hyperexcitability in a mouse model of Alzheimer’s disease. Neuron.

[98] Sanchez-Varo R, Sanchez-Mejias E, Fernandez-Valenzuela JJ, DeCastro V, Mejias-Ortega M, Gomez-Arboledas A, et al. Plaque-associated oligomeric amyloid-beta drives early synaptotoxicity in APP/PS1 mice hippocampus: ultrastructural pathology analysis. Front Neurosci. 2021; 15. 10.3389/fnins.2021.752594. 10.3389/fnins.2021.752594 PMC8600261 34803589

[99] Unger MS, Marschallinger J, Kaindl J, Höfling C, Rossner S, Heneka MT (2016). Early changes in hippocampal neurogenesis in transgenic mouse models for Alzheimer’s disease. Mol Neurobiol.

[100] Nasrabady SE, Rizvi B, Goldman JE, Brickman AM (2018). White matter changes in Alzheimer’s disease: a focus on myelin and oligodendrocytes. Acta Neuropathol Commun.

[101] Acharjee S, Verbeek M, Gomez CD, Bisht K, Lee B, Benoit L (2018). Reduced microglial activity and enhanced glutamate transmission in the basolateral amygdala in early CNS autoimmunity. J Neurosci.

[102] Garofalo S, Porzia A, Mainiero F, Di Angelantonio S, Cortese B, Basilico B (2017). Environmental stimuli shape microglial plasticity in glioma. Elife.

[103] Alvarez-Vergara MI, Rosales-Nieves AE, March-Diaz R, Rodriguez-Perinan G, Lara-Ureña N, Ortega-de SanLuis C (2021). Non-productive angiogenesis disassembles Aβ plaque-associated blood vessels. Nat Commun.

[104] Oe Y, Baba O, Ashida H, Nakamura KC, Hirase H (2016). Glycogen distribution in the microwave-fixed mouse brain reveals heterogeneous astrocytic patterns. Glia.

[105] Guma E, Bordeleau M, González Ibáñez F, Picard K, Snook E, Desrosiers-Grégoire G (2022). Differential effects of early or late exposure to prenatal maternal immune activation on mouse embryonic neurodevelopment. Proc Natl Acad Sci USA.

[106] Hui CW, St-Pierre A, El Hajj H, Remy Y, Hébert SS, Luheshi GN (2018). Prenatal immune challenge in mice leads to partly sex-dependent behavioral, microglial, and molecular abnormalities associated with schizophrenia. Front Mol Neurosci.

[107] Rizou SV, Evangelou K, Myrianthopoulos V, Mourouzis I, Havaki S, Athanasiou A (2019). A novel quantitative method for the detection of lipofuscin, the main by-product of cellular senescence, in fluids. Methods Mol Biol.

[108] Clayton K, Delpech JC, Herron S, Iwahara N, Ericsson M, Saito T (2021). Plaque associated microglia hyper-secrete extracellular vesicles and accelerate tau propagation in a humanized APP mouse model. Mol Neurodegener.

[109] Gyoneva S, Swanger SA, Zhang J, Weinshenker D, Traynelis SF (2016). Altered motility of plaque-associated microglia in a model of Alzheimer’s disease. Neuroscience.

[110] Shukla AK, McIntyre LL, Marsh SE, Schneider CA, Hoover EM, Walsh CM (2019). CD11a expression distinguishes infiltrating myeloid cells from plaque-associated microglia in Alzheimer’s disease. Glia.

[111] Gomez-Arboledas A, Davila JC, Sanchez-Mejias E, Navarro V, Nuñez-Diaz C, Sanchez-Varo R (2018). Phagocytic clearance of presynaptic dystrophies by reactive astrocytes in Alzheimer’s disease. Glia.

[112] Picca A, Calvani R, Coelho-Junior HJ, Landi F, Bernabei R, Marzetti E (2020). Mitochondrial dysfunction, oxidative stress, and neuroinflammation: intertwined roads to neurodegeneration. Antioxidants (Basel).

[113] Zeineh MM, Chen Y, Kitzler HH, Hammond R, Vogel H, Rutt BK (2015). Activated iron-containing microglia in the human hippocampus identified by magnetic resonance imaging in Alzheimer disease. Neurobiol Aging.

[114] Uranova NA, Vikhreva OV, Rakhmanova VI, Orlovskaya DD (2018). Ultrastructural pathology of oligodendrocytes adjacent to microglia in prefrontal white matter in schizophrenia. NPJ Schizophr.

