# Supplementary figures and images for: Ultrastructural characterization of dark microglia during aging in a mouse model of Alzheimer’s disease pathology and in human post-mortem brain samples
Source: J Neuroinflammation. 2022 Sep 27;19:235. doi: 10.1186/s12974-022-02595-8 (PMC9513936; doi:10.1186/s12974-022-02595-8)

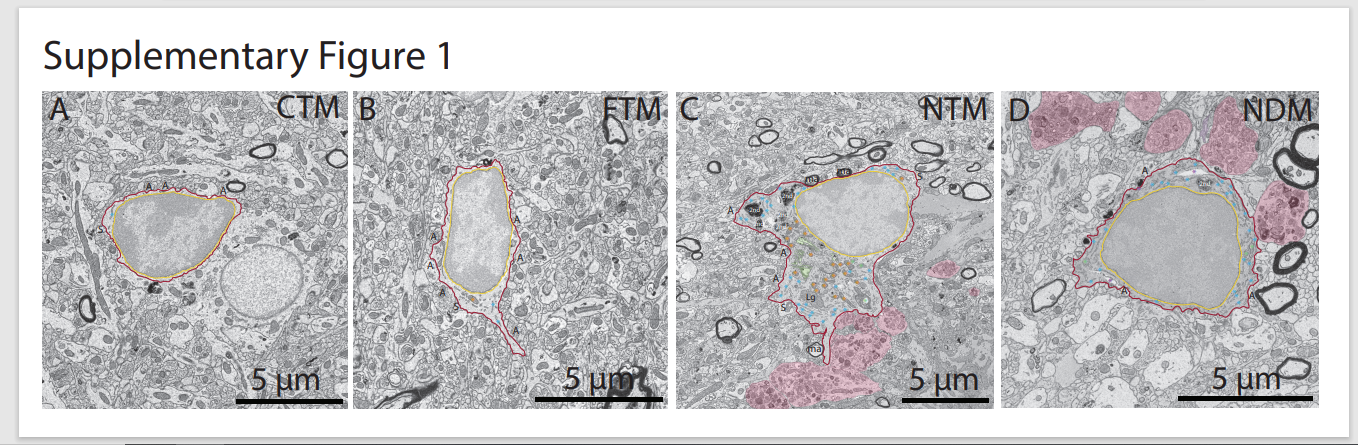

Supplement: Supplementary file 1 — Additional file 1: Fig S1 Presence of dark vs typical microglia in 8-month-old APP-PS1 mice. Representative 5 nm resolution of scanning electron microscopy images captured in the ventral hippocampus CA1 stratum lacunosum-moleculare of 8-month-old APP-PS1 and C57BL/6J mice. (A) typical microglia (TM) in C57BL/6J mice interacting with axon terminals (labeled A) and dendritic spines (labeled S), (B) TM far from a plaque interacting with synaptic elements, (C) TM near plaques with dilated endoplasmic reticulum cisternae (purple asterisk), juxtaposing synaptic elements and dystrophic neurites (pseudocolored in pink), (D) dark microglia (DM) near Aβ plaques with dilated endoplasmic reticulum cisternae and interacting with non-dystrophic and dystrophic synaptic elements in APP-PS1 mice. Red outline = plasma membrane, yellow outline = nuclear membrane. ma = myelinated axons, A = axon terminals, orange asterisk = mitochondria, green asterisk = altered mitochondria, blue asterisk = endoplasmic reticulum, purple asterisk = dilated endoplasmic reticulum, 2rd = tertiary lysosome, pink pseudo-coloring = dystrophic neurites, purple pseudo-coloring = fibrillar Aβ. [file 12974_2022_2595_MOESM1_ESM.png]
